# Hepatocyte programmed cell death: the trigger for inflammation and fibrosis in metabolic dysfunction-associated steatohepatitis

**DOI:** 10.3389/fcell.2024.1431921

**Published:** 2024-07-12

**Authors:** Zilu Cheng, Huikuan Chu, Ekihiro Seki, Rong Lin, Ling Yang

**Affiliations:** ^1^ Division of Gastroenterology, Union Hospital, Tongji Medical College, Huazhong University of Science and Technology, Wuhan, Hubei, China; ^2^ Karsh Division of Gastroenterology and Hepatology, Cedars-Sinai Medical Center, Los Angeles, CA, United States; ^3^ Samuel Oschin Comprehensive Cancer Institute, Cedars-Sinai Medical Center, Los Angeles, CA, United States

**Keywords:** programmed cell death, metabolic dysfunction-associated steatohepatitis, metabolic disorders, hepatic inflammation, hepatic fibrosis

## Abstract

By replacing and removing defective or infected cells, programmed cell death (PCD) contributes to homeostasis maintenance and body development, which is ubiquitously present in mammals and can occur at any time. Besides apoptosis, more novel modalities of PCD have been described recently, such as necroptosis, pyroptosis, ferroptosis, and autophagy-dependent cell death. PCD not only regulates multiple physiological processes, but also participates in the pathogenesis of diverse disorders, including metabolic dysfunction-associated steatotic liver disease (MASLD). MASLD is mainly classified into metabolic dysfunction-associated steatotic liver (MASL) and metabolic dysfunction-associated steatohepatitis (MASH), and the latter putatively progresses to cirrhosis and hepatocellular carcinoma. Owing to increased incidence and obscure etiology of MASH, its management still remains a tremendous challenge. Recently, hepatocyte PCD has been attracted much attention as a potent driver of the pathological progression from MASL to MASH, and some pharmacological agents have been proved to exert their salutary effects on MASH partly via the regulation of the activity of hepatocyte PCD. The current review recapitulates the pathogenesis of different modalities of PCD, clarifies the mechanisms underlying how metabolic disorders in MASLD induce hepatocyte PCD and how hepatocyte PCD contributes to inflammatory and fibrotic progression of MASH, discusses several signaling pathways in hepatocytes governing the execution of PCD, and summarizes some potential pharmacological agents for MASH treatment which exert their therapeutic effects partly via the regulation of hepatocyte PCD. These findings indicate that hepatocyte PCD putatively represents a new therapeutic point of intervention for MASH.

## 1 Introduction

In the past, nonalcoholic liver disease (NAFLD) refers to a continnum of metabolic conditions in the liver with aberrant lipid accumulation in hepatocytes as its primary characteristics, encompassing nonalcoholic fatty liver (NAFL) and nonalcoholic steatohepatitis (NASH) (Younossi et al., 2018). However, owing to the increasingly prominent shortcomings of diagnostic criteria for exclusive diseases and disease terminology with stigmatization issues, NAFLD is recommended to be renamed metabolic dysfunction-associated steatotic liver disease (MASLD) (Fan and Li, 2023; Rinella and Sookoian, 2023). Moreover, NAFL and NASH are also suggested to be replaced with metabolic dysfunction-associated steatotic liver (MASL) metabolic dysfunction-associated steatohepatitis (MASH), respectively (Fan and Li, 2023; Rinella and Sookoian, 2023). Thus, in this review, we unanimously adopt new relevant terminology. With an increasing prevalence of obesity globally, the number of individuals suffered from MASLD is soaring year by year, which accounts for 25% of the world’s population (Diehl et al., 2019). Undoubtedly, the pandemic of MASLD has placed a considerable burden on the social economy and public health (Younossi et al., 2018). However, the etiology of MASLD has hitherto remained intricate and ambiguous, which is implicated in a constellation of drivers, including genetic predisposition, metabolic disorders, oxidative stress, mitochondrial dysfunction, and inflammatory insults (Tilg and Moschen, 2010). MASL is regarded as a benign condition, and most of patients in such a group are less likely to undergo further clinical progression, while more than one third of MASH patients putatively advance to fibrosis, cirrhosis, and hepatocellular carcinoma (HCC) (Calzadilla Bertot and Adams, 2016; Marengo et al., 2016). Thus, it is of paramount importance to identify critical drivers which expedite the progression from MASL to MASH (Calzadilla Bertot and Adams, 2016; Koyama and Brenner, 2017), and the ablation of these drivers probably represents an effective therapeutic avenue for MASH.

Cell death is a ubiquitous and irreversible life phenomenon, which shows an intimate correlation with diverse biological and pathological processes (Green, 2022). Programmed cell death (PCD) belongs to an important and common modality of cell death, which is defined as an active cell death procedure response to multiple biological and pathological stimulations (Bedoui et al., 2020). For many years in the past, PCD has been synonymous with apoptosis, which played an indispensable role in modulating homeostasis maintenance and body development (Griffioen and Nowak-Sliwinska, 2022). With more intensive exploration of PCD, many other modalities of PCD have been gradually described, including necroptosis, pyroptosis, autophagy-dependent cell death as well as ferroptosis (Griffioen and Nowak-Sliwinska, 2022). Each form of PCD possesses its distinct molecular, biochemical, and morphological characteristics, and exerts different physiological effects (Gautheron et al., 2020). Recently, a growing body of studies have suggested that the metabolic alterations in MASLD acted as a potent trigger for hepatocyte PCD, which further elicited a series of inflammatory and fibrotic responses, greatly contributing to the transition of MASL to MASH (Feldstein et al., 2003; Nan et al., 2007; Volkmann et al., 2007; Hirsova et al., 2013).

Restricted calorie intake and proper exercise are deemed to be the predominant therapeutic approaches for the early stage of MASLD, but when the disease advances to MASH and cirrhosis, single lifestyle intervention is insufficient (Diehl et al., 2019). MASH management still remains a tremendous challenge, which highly demands the emergence of new effective therapies. With consideration of the pivotal role of PCD in MASH pathogenesis, it is highly necessary to elucidate the mechanisms underlying the impact of PCD on MASH, which contributes to find novel therapeutic point of interventional strategies for MASH.

## 2 Pathogenesis of PCD

### 2.1 Apoptosis

**FIGURE 1:**
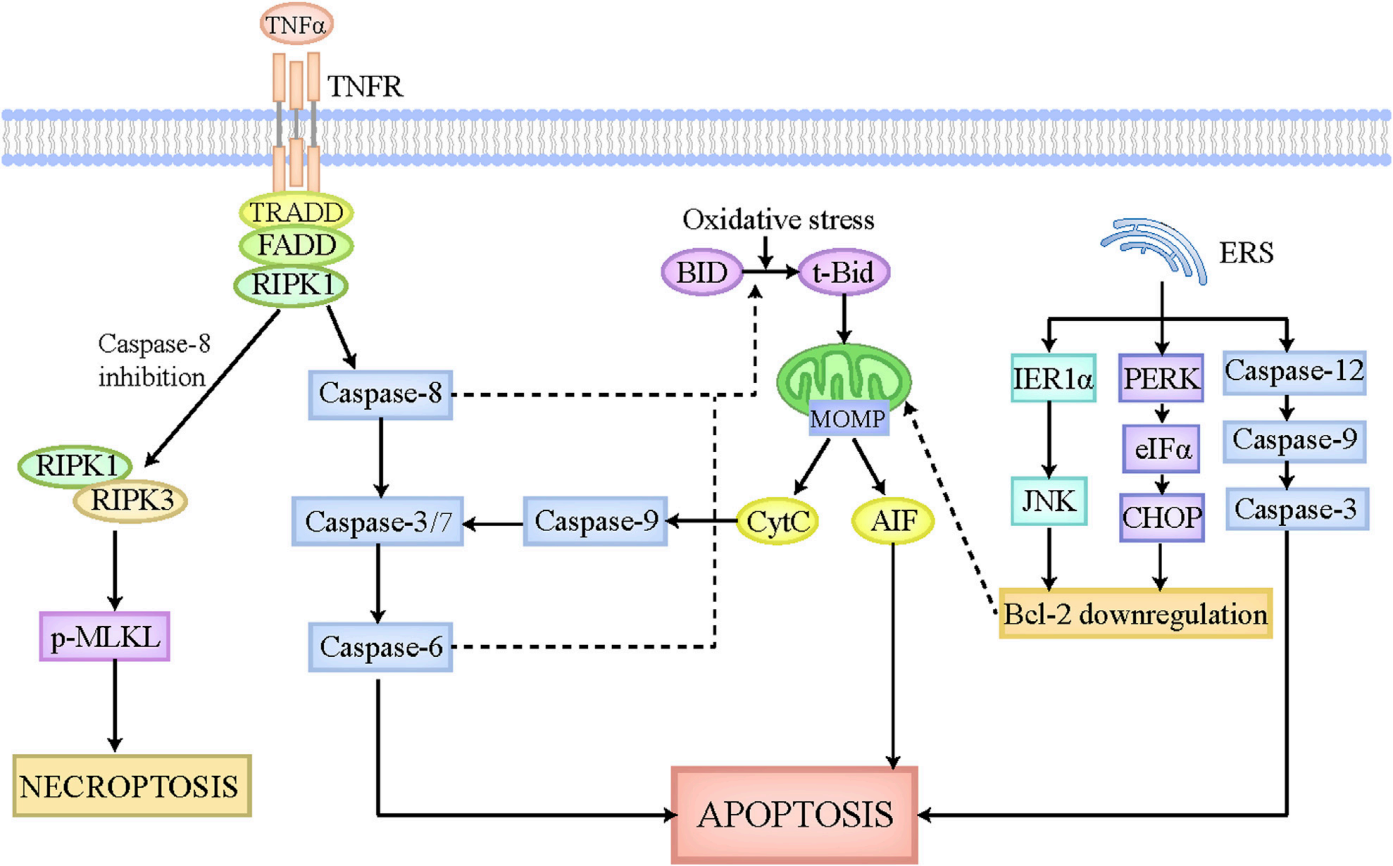
The pathophysiology of apoptosis and necroptosis. The apoptotic pathway can be executed by death receptor pathway, mitochondrial pathway, and endoplasmic reticulum pathway. The death receptors located on cellular membrane can be recognized and activated by death ligands, such as FasL and TNFα, which then is able to recruit associated adaptor proteins and assemble the death-inducing signaling complex. This complex leads to the activation of caspase-8, which can activate caspase-3/7 and caspase-6 sequentially, ultimately causing apoptosis. Intrinsic stimulations like oxidative stress cleaves BID to tBid, which can translocate to the mitochondria where it induces MOMP formation and CytC and AIF release. The released CytC activates caspase-9 and caspase-3/7 sequentially, ultimately leading to apoptosis. Moreover, in hepatocytes, caspase-8 and caspase-6 also participate in the cleavage of tBid and the release of CytC, thus strikingly amplifying the death signal initiated by death receptors. ERS also plays a critical role in apoptosis execution. On the one hand, ERS initiates the mitochondrial apoptosis reaction chain by downregulating Bcl-2 via IRE1α-JNK and PERK-eIFα-CHOP axis. On the other hand, ERS activates caspase-12, caspase-9, and caspase-3 sequentially, culminating in apoptosis. When capase-8 is inhibited, the death-inducing signaling complex mentioned above activates RIPK1, which then recruits RIPK3 and phosphorylates MLKL. The phosphorylated MLKL is then translocated to cellular membrane where it undergoes oligomerization and triggers necroptosis. Abbreviations: TNF, tumor necrosis factor; MOMP, mitochondrial outer membrane pore formation; BID, BH3-interacting domain death agonist; CytC, Cytochrome c; ERS, endoplasmic reticulum stress; RIPK1, receptor-interacting protein kinase-1; MLKL, pseudokinase mixed lineage domain-like.

Apoptosis is identified as an important and universal physiological cell death regulated by multiple signaling pathways and genes, which aims to maintain cellular homeostasis when exposing to diverse intrinsic and extrinsic stimulations. The morphological characteristics of apoptosis are implicated in cellular shrinkage, chromatin agglutination, and apoptotic bodies formation (Van Cruchten and Van Den Broeck, 2002). The apoptotic bodies can be degraded and removed by adjacent cells or macrophages, which scarcely elicits inflammatory responses without the leakage of cellular components (Kerr et al., 1972). Apoptosis is executed by extrinsic pathway and intrinsic pathway (Figure 1). In extrinsic apoptosis, death ligands like FasL and tumor necrosis factor (TNF), initiate the activation of death receptors located on cellular membrane, and then recruit their associated adaptor proteins, such as receptor-interacting protein kinase-1 (RIPK1) and TNF receptor-associated factors 2 or 5, to assemble the death-inducing signaling complex, which has the capacity to activate caspase-8 and caspase-3/7 sequentially (Galluzzi et al., 2018). Caspase-3/7 activate caspase-6 and then trigger the execution of apoptosis (Galluzzi et al., 2018). In contrast, the mitochondria play a major role in intrinsic apoptosis, which is regulated by B-cell lymphoma-2 (Bcl-2) family to contribute to apoptosis pathogenesis (Alkhouri et al., 2011). The Bcl-2 family is mainly comprised of antiapoptotic members (Mcl-1, Bcl-xL, Bcl-2, etc.) and proapoptotic members (Bim, Bid, Bak, Bax, etc.) (Alkhouri et al., 2011). Under the physiological status, Bcl-2 combines with Bax to constitute a heterodimer, which inhibits mitochondrial outer membrane pore formation (MOMP). When cells are exposed to intrinsic stimulations like oxidative stress, the BH3-interacting domain death agonist (BID), an activator of Bcl-2, is cleaved and transformed to t-Bid, which can translocate to the mitochondria where it leads to conformation changes of mitochondrial outer membrane pore through interacting with Bak and Bax, ultimately bringing about MOMP and the release of apoptotic inducing factors, including Cytochrome c (CytC) (Galluzzi et al., 2018). The cytoplasmic CytC triggers caspase-9 activation, which has the capacity to activate caspase-3/7, ultimately inducing apoptosis (Galluzzi et al., 2018). Of note, in hepatocytes, tBid formation and its subsequent translocation into mitochondria are essential for extrinsic apoptosis, which strikingly amplifies the death signal evoked by death ligands (Iorga et al., 2017). Caspase-8 and caspase-6 in hepatocytes have been reported to be responsible for the cleavage of tBid and the release of CytC (Zhao et al., 2020). Moreover, endoplasmic reticulum stress (ERS) has recently emerged as another pathway to induce apoptosis. On the one hand, ERS initiates the mitochondrial apoptosis reaction chain by downregulating Bcl-2 via IRE1α-JNK and PERK-eIFα-CHOP axis (Malhi and Kaufman, 2011; Oakes and Papa, 2015). On the other hand, ERS activates caspase-12, caspase-9, and caspase-3 sequentially, culminating in apoptosis (Malhi and Kaufman, 2011).

### 2.2 Necroptosis

Necroptosis is referred to one kind of lytic cell death which shares the common upstream activation signaling pathways with apoptosis (Han et al., 2011). But intriguingly, the morphological features of necroptosis are not similar to apoptosis, but similar to necrosis, which mainly characterize cell swelling, membranolysis, as well as the leakage of cellular constituents (Pasparakis and Vandenabeele, 2015). The released cellular components, including damaged associated molecular patterns (DAMPs), cytokines, and chemokines, have the capacity to potently trigger inflammatory cascades (Pasparakis and Vandenabeele, 2015). Generally, the occurrence of necroptosis requires death receptors activation and apoptosis inhibition (Figure 1). In the context of caspase-8 paucity, deubiquitinase cylindricin activates RIPK1, which then interacts with RIPK3 via their common RIP homology interaction motif (Sun et al., 2012). Subsequently, RIPK3 recruits the pseudokinase mixed lineage domain-like (MLKL) and makes it phosphorylated and activated. The phosphorylated MLKL is then translocated to cellular membrane where it undergoes oligomerization and triggers necroptosis (Sun et al., 2012; Murphy et al., 2013). The mechanisms by which p-MLKL induces necroptosis are described below. On the one hand, p-MLKL acts as a platform for recruiting sodium ions or calcium ions in the cellular membrane, thus affecting the intracellular and extracellular osmotic pressure, and ultimately leading to the swelling of cells and the rupture of cellular membrane (Dhuriya and Sharma, 2018). On the other hand, p-MLKL is able to interact with the amino terminal of sphingomyelin phosphoinositide distributed in cellular membrane and further promote the formation of cellular membrane pores, finally causing the leakage of cellular constituents (Dhuriya and Sharma, 2018). Furthermore, there still exist many other signaling pathways governing the execution of necroptosis, which are independent of RIPK1, such as adhesion receptors, transmembrane protein 173, retinoic acid inducible gene 1 protein, nucleic acid sensors and so on (Schock et al., 2017; Brault et al., 2018; Upton et al., 2019). But notably, these pathways also require RIPK3 to phosphorylate and activate MLKL, ultimately causing necroptosis.

### 2.3 Pyroptosis

**FIGURE 2:**
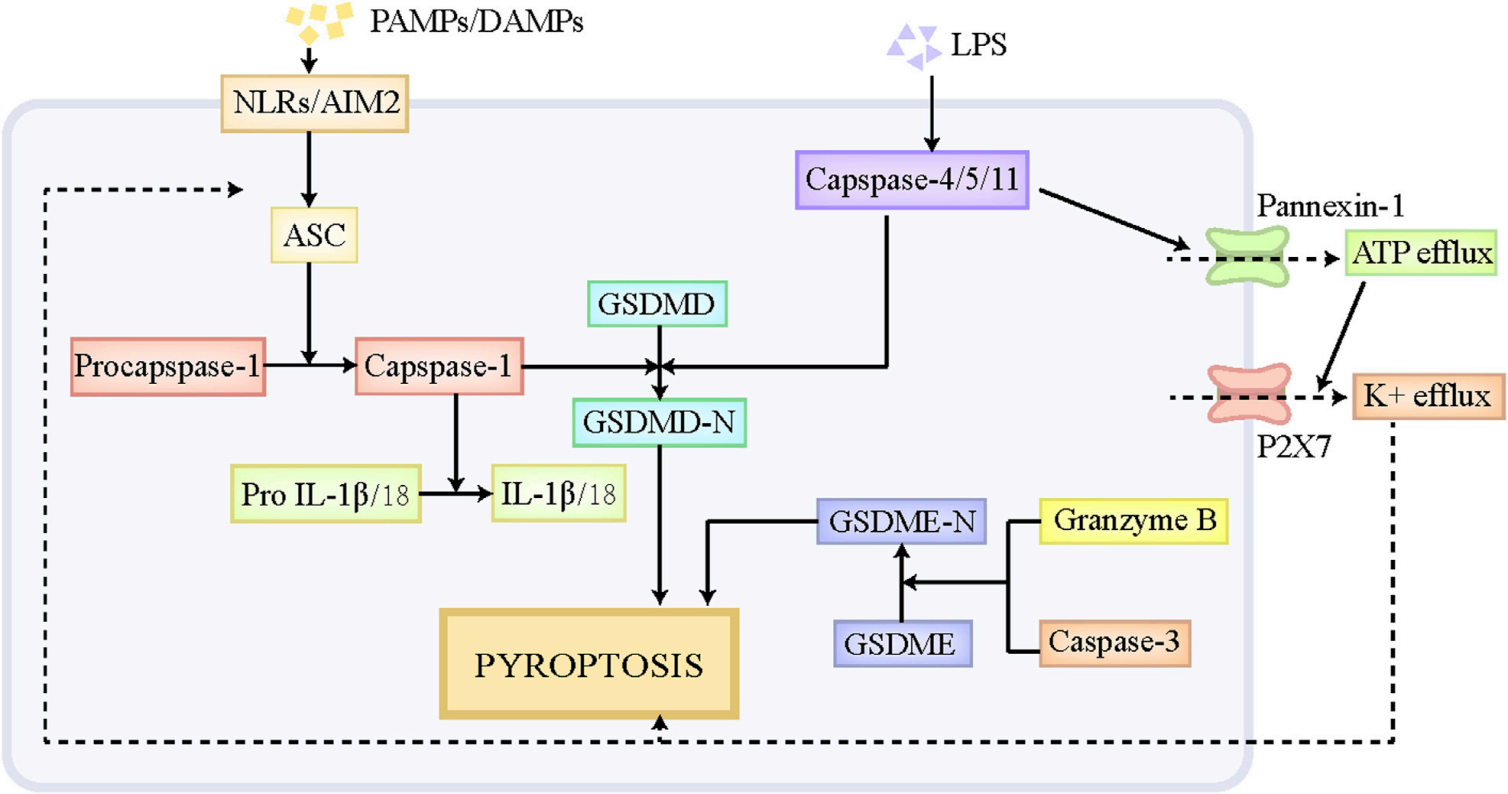
The pathophysiology of pyroptosis. NLRs and AIM2 can recognize endogenous DAMPs and exogenous PAMPs, and then recruits pro-capase-1 by interacting with ASC, thus forming an inflammasome, which cleaves pro-caspase-1 to caspase-1. Caspase-1 subsequently leads to the cleavage of GSDMD and releases its N-terminal fragment, namely GSDMD-N, which ultimately results in pyroptosis. Pro-caspase-4/5/11 has the capacity to directly bind to liposome A at the tail of LPS, which promotes the self-oligomerization and self-activation of caspase-4/5/11. The activated caspase-4/5/11 is able to cleave GSDMD to GSDMD-N and induce pyroptosis. Moreover, Pannexin-1, the channel protein on cellular membrane, also can be cleaved by capsase-11, which then mediates the efflux of ATP. The repeated stimulation of ATP is a potent driver for P2X7 channel opening, which triggers the efflux of K^+^, Ca^+^, and Na^+^, thus causing cell swelling and lytic cell death. In addition, K^+^efflux also serves as a contributor to activate NLRP3/ASC/caspase-1 signaling axis, further promoting the cleavage of GSDMD. Besides GSDMD, GSDME also play roles in the pathogenesis of pyroptosis. GSDME is cleaved to GSDME-N by granzyme B and caspase-3, which similarly induces the pore formation in cellular membrane and ultimate pyroptosis. Abbreviations: DAMPs, damaged associated molecular patterns; PAMPs, pathogen-associated molecular patterns; NLRs, Nod-like receptors; AIM2, absent in melanoma-2; GSDMD, gasdermin D.

Pyroptosis is a special form of PCD, which has similar morphological characteristics with necroptosis, including cell swelling, membranolysis, as well as the leakage of cellular components (de Vasconcelos et al., 2019). Moderate pyroptosis contributes to the removal of pathogen-infected and defective cells by the immune system, while excessive pyroptosis putatively exacerbates inflammatory responses and causes massive cell death and tissue injury (Sharma and Kanneganti, 2021). Pyroptosis participates in multiple disorders, including central nervous system diseases, infectious disorders, liver diseases and so on (Tricarico et al., 2013; Zhou et al., 2018). The execution of pyroptosis is primarily regulated through the canonical pathway mediated by caspase-1 and the noncanonical pathway mediated by caspase-11 (Figure 2). The classical pyroptotic pathway is initiated by diverse pathogen-associated molecular patterns (PAMPs) and DAMPs, and mainly depends on the assembly of inflammasome to execute cell death (Amarante-Mendes et al., 2018). The inflammasome is comprised of the sensor, Nod-like receptors (NLRs) and absent in melanoma-2 (AIM2), the bridge adaptor, apoptosis-associated speck-like protein containing a caspase recruitment domain (ASC), as well as the effector, pro-caspase-1 (Man et al., 2017). NLRs and AIM2 firstly recognizes endogenous DAMPs and exogenous PAMPs, and then recruits pro-caspase-1 by interacting with ASC, thus forming an inflammasome, which cleaves pro-caspase-1 to generate caspase-1 (Xia et al., 2019). Caspase-1 subsequently leads to the cleavage of the protein gasdermin D (GSDMD) and releases its N-terminal fragment, namely GSDMD-N, which ultimately results in the pore formation in the cellular membrane and the leakage of cellular contents (Xia et al., 2019). Moreover, caspase-1 also facilitates the maturation of interleukin (IL)-18 and IL-1β and triggers the release of these inflammatory cytokines, which further contributes to the amplification of the inflammatory signal (Bani-Hani et al., 2009). In noncanonical pyroptosis, pro-caspase-4/5/11 has the capacity to directly bind to lipid A at the tail of lipopolysaccharide (LPS), which promotes the self-oligomerization and self-activation of caspase-4/5/11. The activated caspase-4/5/11 then cleaves GSDMD to GSDMD-N and induce pyroptosis (Wu J. et al., 2021). Moreover, Pannexin-1, the channel protein on cellular membrane, is cleaved by capsase-11, which then mediates the efflux of ATP (Yang et al., 2015). The repeated stimulation of ATP is a potent driver for P2X7 channel opening, which triggers the efflux of K^+^, Ca^+^, and Na^+^, thus causing cell swelling and lytic cell death (Yang et al., 2015). In addition, K^+^efflux also serves as a contributor to activate NLRP3/ASC/caspase-1 signaling axis, further promoting the release of IL-18 and IL-1β (Xia et al., 2019; Wang et al., 2020). Besides GSDMD, GSDME and GSDMB also play roles in the pathogenesis of pyroptosis (Rogers et al., 2017; Kambara et al., 2018; Chen et al., 2019). Mechanistically, GSDME is cleaved to GSDME-N by granzyme B and caspase-3, which also induces the pore formation in cellular membrane and ultimate pyroptosis (Rogers et al., 2017; Kambara et al., 2018). Notably, caspase-3 also participates in apoptotic pathway, and the specific form of cell death that caspase-3 mediates mainly depends on the expression level of GSDME in cells. The high level of GSDME leads to the transition from caspase-3-induced apoptosis to caspase-3-induced pyroptosis. Different from GSDMD-N and GSDME-N, GSDMB-N is unable to trigger cell death directly (Chen et al., 2019). GSDMB-N contributes to noncanonical pyroptotic pathway by binding and activating caspase-4 (Chen et al., 2019).

### 2.4 Autophagy and autophagy-dependent cell death

**FIGURE 3:**
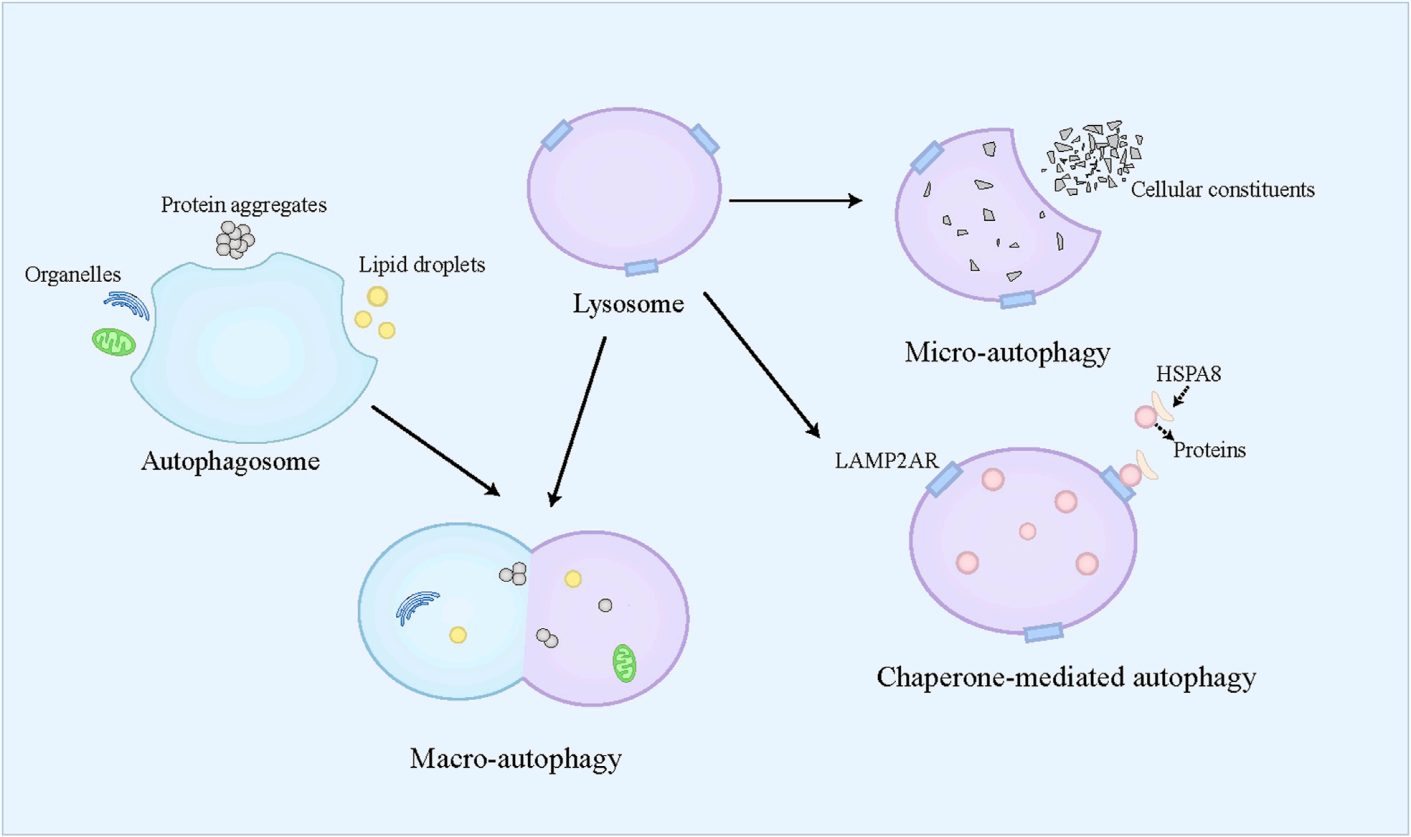
An overview of autophagy. Autophagy is classified into three modalities, namely macro-autophagy, micro-autophagy, and chaperone-mediated autophagy. Macro-autophagy requires autophagosome formation, which can selectively engulf and sequester part of the cellular contents, such as lipid droplets, protein aggregates, and organelles. Then, the autophagosome is fused with the lysosome, where these cellular contents are degraded. Micro-autophagy refers to the direct engulfment and subsequent degradation of cellular constituents by the lysosome. Chaperone-mediated autophagy is specialized in degrading the proteins containing the KFERQ-like motif, which combine with HSPA8, and then bind to LAMP2A receptor on the lysosome and be further translocated to the lysosome where these proteins are degraded.

Autophagy is referred to a self-eating catabolic process relying on the lysosome, which is extensively present in eukaryotic cells. Autophagy not only facilitates the reutilization of cellular substances and provides energy for cell survival by degrading cellular aged and damaged organelles, misfolded proteins, and other molecules, but also exerts protective effects on cells by counteracting oxidative stress (Buzun et al., 2021). Autophagy dysfunction contributes to the pathogenesis of diverse conditions, including neurodegenerative disorders, cancers, and metabolic diseases (Jiang and Mizushima, 2014). Generally, autophagy is classified into three modalities, namely macro-autophagy, micro-autophagy, and chaperone-mediated autophagy (CMA) (Figure 3). Macro-autophagy is widely recognized as the primary form of autophagy and responsible for the degradation of organelles and microorganisms (Zhao et al., 2022). Macro-autophagy requires autophagosome formation, which belongs to a double-membrane vesicle (Dikic and Elazar, 2018). The autophagosome can selectively engulf and sequester part of the cellular contents, such as lipid droplets, protein aggregates, and organelles, which are also called cargoes (Allaire et al., 2019). These cargoes can be further delivered to the lysosome for degradation (Kast and Dominguez, 2017). Micro-autophagy refers to the direct engulfment and subsequent degradation of cellular constituents by the lysosome (Filali-Mouncef et al., 2022). Recently, a kind of micro-autophagy, endosomal micro-autophagy has been described in mammal animals (Oku and Sakai, 2018). The endosomal micro-autophagy recognizes and engulfs the proteins containing the KFERQ-like motif with the auxiliary of HSPA8, and then translocates these proteins to the multivesicular bodies and late endosomal dependent of ESCRT-III (Mejlvang et al., 2018). Finally, the translocated proteins in the multivesicular bodies and late endosomal are delivered to the lysosome to degrade (Tekirdag and Cuervo, 2018). CMA is specialized in degrading the proteins containing the KFERQ-like motif (Kaushik and Cuervo, 2012). Different from endosomal micro-autophagy, the lysosome directly captures the targeted proteins (Kaushik and Cuervo, 2012). The targeted proteins combine with HSPA8 and its cochaperones to form a complex, which can bind to LAMP2A receptor on the lysosome and be further translocated to the lysosome where these proteins are degraded (Cuervo and Dice, 1996; Kaushik and Cuervo, 2012; Kaushik and Cuervo, 2018). In most cases, autophagy belongs to a survival process. However, when the cell cannot overcome lethal stress, the dysregulated autophagy leads to cell death, namely autophagic cell death, which is often concomitant with large-scale autophagic vacuolization in cytoplasm (Kroemer and Levine, 2008). Recently, “autophagy-dependent cell death” has been used to define the modality of cell death caused by autophagy (Galluzzi et al., 2018). Autophagy-dependent death plays an important role in mammalian embryogenesis, and induces cell death in the apoptosis-resistant cell (Baehrecke, 2003; Shimizu et al., 2004). The simultaneous activation of autophagy and JNK has been identified as the prerequisite for autophagy-dependent death (Baehrecke, 2003; Shimizu et al., 2014).

### 2.5 Ferroptosis

Ferroptosis has been described in recent years as a new modality of PCD marked by iron accumulation and overwhelming lipid peroxidation (Dixon et al., 2012). The morphological features of ferroptosis encompass mitochondrial atrophy, decreased or obliterated mitochondrial cristae, as well as cellular membrane rupture (Gautheron et al., 2020). Owing to the leakage of cellular components, ferroptosis also induces a series of inflammatory and immune responses, thus taking an important part in the pathogenesis of diverse diseases, especially liver disorders (Gautheron et al., 2020). The execution of ferroptosis is mainly driven by toxic lipid peroxides and ROS, which can be produced in the process of lipid peroxidation. Of note, polyunsaturated fatty acids (PUFAs) serve as critical substrates for lipid peroxidation, which take part in the development of ferroptosis only in the form of PUFA-PL (Jia et al., 2021). The esterification of PUFA to PUFA-PL is mainly dependent on the catalyzation of lysophosphatidylcholine acyltransferase 3 (LPCAT3) and Acyl-CoA synthetase long-chain family member 4 (ACSL4) (Kagan et al., 2017). Subsequently, lipoxygenases oxidize PUFA-PL to PUFA-PLOOH, which has the capacity to induce ferroptosis (Haeggström and Funk, 2011). In addition, free iron, mainly in the form of Fe^2+^, also contributes to the pathological progression of lipid peroxidation (Berson et al., 2004). Excessive cellular iron firstly reacts with ROS derived from the mitochondria, and then leads to the generation of OH* through the Fenton reaction (Zhang H. et al., 2021). OH* can convert PUFA-PL to PUFA-PL* by abstracting hydrogen from PUFA-PL, and PUFA-PL* is susceptible to O_2_ and is readily transformed into PUFA-PLOO*, which interacts with adjacent PUFA-PL to generate PUFA-PLOOH and next PUFA-PL*, thus inducing the propagation of lipid peroxidation and subsequent ferroptosis (Haeggström and Funk, 2011). Of note, the insights into potential mechanisms underlying how lipid peroxidation induces ferroptosis remain scarce, which warrants further investigations.

## 3 Metabolic alterations trigger hepatocyte PCD

### 3.1 Lipotoxicity

Lipotoxicity is a cardinal feature of MASLD, which shows a strong correlation with organelle dysfunction, cell damage and death, expediting the pathological transition from MASL to MASH. Free fatty acids (FFAs), triglycerides (TGs), ceramides, lysophosphatidylcholine (LPC), and free cholesterol (FC) are accumulated in the hepatocytes of MASH (Yamada et al., 2015). Intriguingly, TGs in hepatocytes are known to protect against cell toxicity, while FFAs, especially saturated FFAs, can be a trigger for lipotoxicity (Listenberger et al., 2003). *In vitro*, palmitate treatment activates the death receptor, TRAIL-R2, which initiates hepatocyte apoptosis in a caspase-8-dependent manner (Cazanave et al., 2011). Caspase-8 not only plays an essential role in extrinsic apoptosis, but also engages in mitochondrial apoptosis to amplify the death signal triggered by death receptors. In the livers of MASH in animals and patients, increased TRAIL-R2 expression was observed, and TRAIL-R2 deficient mice showed improved hepatocyte apoptosis (Volkmann et al., 2007; Hirsova et al., 2013). These evidences demonstrate the significance of TRAIL-R2 in lipotoxicity-induced hepatocyte apoptosis. Moreover, chronic lipid overload can be a major activator for ERS, which activates the apoptotic pathways by multiple mechanisms. On the one hand, ERS has the capacity to increase the expression of proapoptotic proteins, including TRAIL-R2, PUMA and Bim (Barreyro et al., 2007; Akazawa et al., 2010; Cazanave et al., 2010). Among which, Bim and PUMA greatly contribute to the mitochondrial apoptotic signaling and subsequently induce apoptosis (Akazawa et al., 2010; Cazanave et al., 2010). On the other hand, ERS also induces cell death by activating caspase-12, caspase-9, and caspase-3 sequentially. In addition, ceramide acts as a crucial driver for apoptosis by activating death receptors, curbing diverse anti-apoptotic signaling transducers, exerting direct mitochondriotoxic effects, as well as promoting ROS production (Cifone et al., 1994; Arora et al., 1997; García-Ruiz et al., 1997). Furthermore, FC and LPC are both intimately related to mitochondrial membrane pore transition, which brings about the interruption of oxidative respiratory chain reaction, ROS generation, and the release of CytC from mitochondria into cytoplasm, culminating in apoptosis (Fuchs and Steller, 2011). Cholesterol crystals can initiate the activation of NLRP3/ASC/caspase-1 signaling axis, contributing to the execution of pyroptosis (Duewell et al., 2010). Notably, although autophagy is increased by the acute lipid stimulation, it can be inhibited by chronic lipid overload (Bernales et al., 2007; Czaja, 2010; Ding et al., 2010). Impaired autophagy has a positive correlation with hepatic lipid accumulation and ERS, which further exacerbates hepatocyte damage and death.

### 3.2 Glucotoxicity

Besides high fat diet (HFD), the consumption of sugary sweetened beverage is also deemed to be a significant risk for MASH. Several studies have suggested that fructose intake contributed to the pathological progression of MASH and showed a positive correlation with liver fibrosis severity in a dose-dependent manner (Ouyang et al., 2008). Excessive consumption of sugary sweetened beverage and insulin resistance (IR) increase systemic glucose level, which exerts detrimental effects on hepatocytes (Lim et al., 2010). This phenomenon is known as glucotoxicity. Fructose is identified as the primary mediator of glucotoxicity. In both animal and human studies, fructose intake was confirmed to promote *de novo* lipogenesis and inhibit fatty acid oxidation in the liver, thus exacerbating lipid accumulation in hepatocytes (Lim et al., 2010; Softic et al., 2016). Herein, it is reasonable to draw a conclusion that fructose probably engages the regulation of cell death by contributing to lipotoxicity. Moreover, fructose can also directly activate caspase-3 and cause PUMA and Bim-mediated mitochondrial dysfunction through activating JNK and upregulating CHOP, ultimately resulting in apoptosis (Zhong et al., 2012; Wali et al., 2014; Jaiswal et al., 2015). In addition, the metabolism of fructose is intimately linked to the production of uric acids, which have multifaceted functions (Cox et al., 2012). Specifically, uric acids are able to aggravate hepatic steatosis by restraining fatty acid oxidation and stimulating gluconeogenesis and lipogenesis, induce mitochondrial oxidative stress by activating NAPDH oxidase and translocating it into mitochondria, and trigger pyroptosis by activating the NLRP3/ASC/caspase-1 signaling axis (Martinon et al., 2006; Lanaspa et al., 2012; Jensen et al., 2018). Furthermore, fructose metabolism in the gut strongly correlates with the disruption of gut tight junctions, which leads to increased intestinal permeability and subsequent translocation of PAMPs from the gut to the liver (Johnson et al., 2013). As mentioned previously, PAMPs, including LPS, are recognized as the major drivers for pyroptosis.

### 3.3 Iron and copper

In one-third of MASLD patients, the metabolic disorders of iron and copper were observed (Aigner et al., 2015). Iron is a vital and common trace element in human body, which not only acts as a critical constituent of diverse essential enzymes, but also takes an important part in the redox system. In a cross-sectional study, dietary iron intake positively correlated with MASLD prevalence dose-dependently (Yang et al., 2019). And aberrant iron accumulation promotes the transition of MASL to MASH by inducing oxidative stress, inflammatory responses, and cell damage or cell death. In recent years, iron accumulation has been emerged as a cardinal hallmark of a novel modality of PCD, namely ferroptosis, which greatly contributes to the development of MASH (Wu S. et al., 2021). As indicated above, iron promotes the generation of toxic lipid peroxides and the propagation of lipid peroxidation, thus triggering the execution of ferroptosis (Berson et al., 2004; Zhang H. et al., 2021). Copper is another essential trace element, which has the capacity to assist massive metalloproteins to form redox active centers (Aigner et al., 2010). Low hepatic copper level was observed in patients with MASLD, which also gave impetus to the development of MASLD by disturbing antioxidant defense systems, promoting hepatic lipid accumulation, and causing iron retention (Aigner et al., 2008; Aigner et al., 2010). Copper also engages the pathogenesis of multiple cell death. Previously, copper was considered to be a potent trigger for apoptosis and autophagy (Kang et al., 2019; Jiang Y. et al., 2022). It could induce apoptosis by generating ROS via the Fenton reaction and upregulating the expression of apoptosis-related genes (Kang et al., 2019). Similarly, copper triggered autophagy by upregulating autophagy-assisted proteins, including p62 and LC3 (Kang et al., 2019). It is worthy to note that a novel copper-dependent PCD has been described in a recent study, which is termed cuproptosis (Tsvetkov et al., 2022). Copper can directly bind lipoylated constituents in tricarboxylic acid cycle, which further induces lipoylated proteins aggregation and subsequent degradation of iron-sulfur cluster proteins, resulting in proteotoxic responses and cell death (Tsvetkov et al., 2022). Whether reduced hepatic copper concentration affects the progression of MASH by modulating hepatocyte cell death remains obscure, demanding the emergence of new evidence.

### 3.4 PAMPs/MAMPs

The dysregulation of gut microbiota intimately correlates with MASH etiology, which leads to compromised gut barrier and increased gut permeability (Safari and Gérard, 2019). High fructose consumption also contributes to increased gut permeability in MASH (Johnson et al., 2013). In this context, gut pathological microorganisms can be translocated into the liver through portal vein. These pathogens release PAMPs and microbe-associated molecular patterns (MAMPs), which can be recognized by PRRs, such as TLRs, and trigger a series of signaling pathways. Intracellular PAMPs can activate NLRs and initiates the assembly of the inflammasome complex mainly comprised of NLRP3, ASC, and pro-caspase-1, which can subsequently cleave pro-caspase-1 to caspase-1 and executes the downstream pyroptotic pathway as mentioned above (Man et al., 2017; Amarante-Mendes et al., 2018; Xia et al., 2019). Moreover, intracellular LPS, as a PAMP, can directly activate caspase-4/5/11 sequentially and then induce pyroptosis (Wu J. et al., 2021).

## 4 Hepatocyte PCD and its related cellular crosstalk induce inflammation, fibrosis in MASH

### 4.1 Hepatocyte apoptosis

**FIGURE 4:**
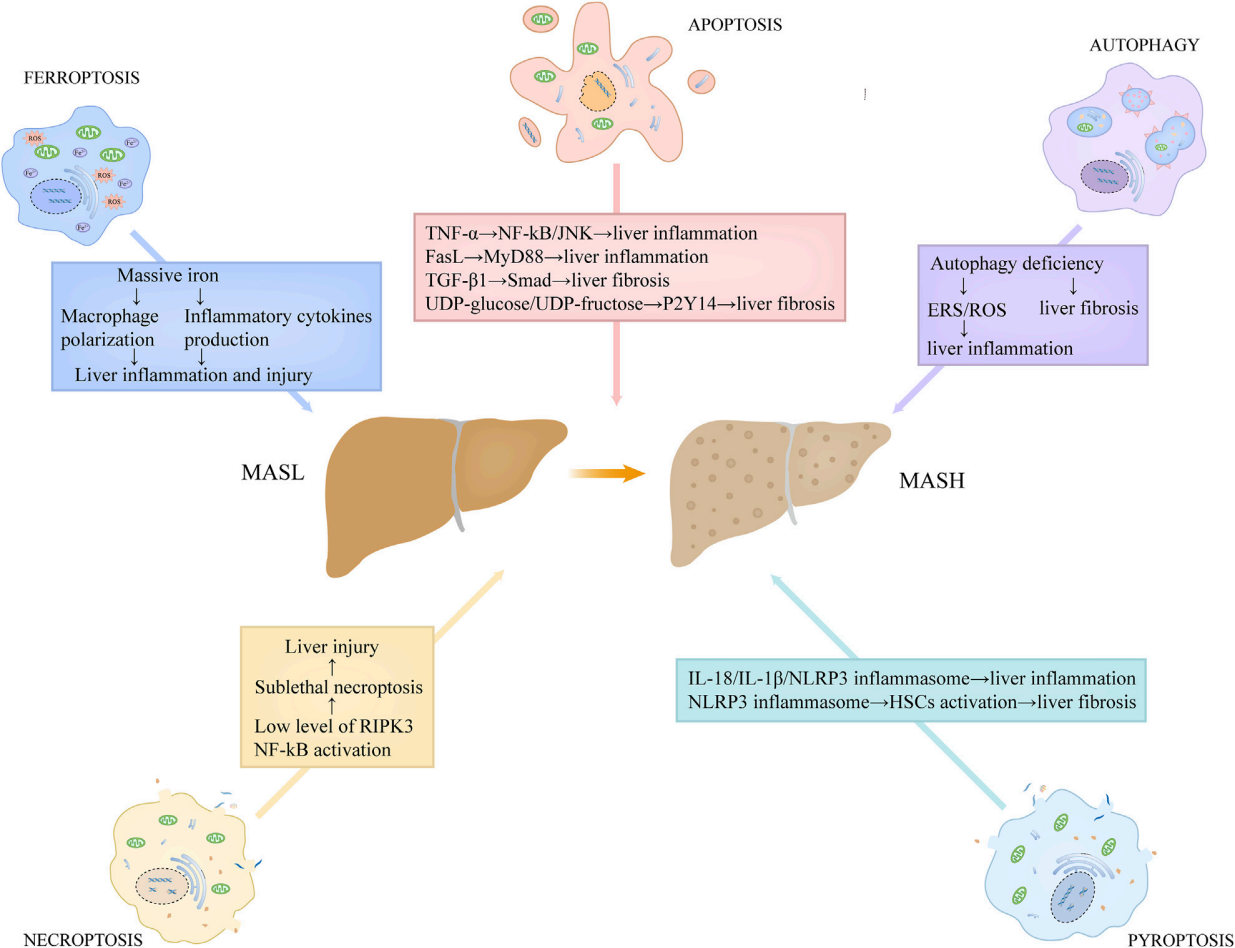
The roles of programmed cell death in the proinflammatory and fibrotic progression in MASH. Hepatic apoptotic bodies derived from apoptotic process stimulate the production of death receptor ligands, such as TNF-α and FasL. TNF-α has the capacity to activate NF-kB and JNK pathways, triggering a series of inflammatory responses. FasL stimulates the secretion of chemokines in macrophages through MyD88 signaling axis. Hepatocyte apoptosis also correlates with the production of TGF-β1 and the release of purinergic ligands, including UDP-glucose and UDP-fructose. Among which, TGF-β1 contributes to liver fibrosis through facilitating extracellular matrix deposition via TGF-β1/Smad axis in HSCs, while UDP-glucose/UDP-fructose recognizes purinergic receptor P2Y14 distributed in HSCs, resulting in the activation of HSCs and liver fibrosis. Low expression of RIPK3 level in hepatocytes and NF-kB activation in MASH may lead to sublethal necroptosis, which expedites the progression of the disease. Pyroptosis induces the release of massive inflammatory cytokines, such as IL-18 and IL-1β, directly promoting inflammatory responses. Moreover, the released NLRP3 inflammasome particles in pyroptosis can be engulfed by HSCs, which results in HSCs activation and α-SMA upregulation, contributing to liver fibrosis. Autophagy deficiency in MASH is unfavorable for the elimination of ERS and dysregulated or impaired mitochondria, thus potentiating ROS generation, which promotes liver inflammation. In addition, hepatocyte autophagy deficiency also contributes to liver fibrosis. Abbreviations: TNF, tumor necrosis factor; MASH, metabolic dysfunction-associated steatohepatitis; HSCs, hepatic stellate cells; NF-kB, nuclear factor-kB; TGF-β1, transforming growth factor-β1; ERS, Endoplasmic reticulum stress; ROS, reactive oxidative stress; RIPK3, receptor-interacting protein kinase 3.

Hepatocyte apoptosis is linked to a variety of liver disorders, including MASLD. On the basis of several recent studies, hepatocyte apoptosis is associated with the progression of inflammation and fibrosis in MASH, a severe modality of MASLD (Figure 4) (Feldstein et al., 2003; Nan et al., 2007). Feldstein and colleagues (Feldstein et al., 2003) demonstrated that, compared to the controls, several apoptosis-related indicators, including the TUNEL positivity, the levels of activated caspase-3/7, and the expression level of death receptors, were increased in the hepatic specimens from MASH patients. They also found that hepatocyte apoptosis showed a positive correlation with the severity of liver inflammation and fibrosis (Feldstein et al., 2003). Moreover, Nan et al. (Nan et al., 2007) used a MASH mouse model by feeding them with methionine-choline deficient (MCD) diet, and found the similar observation that hepatocyte apoptosis was increased in MASH group and that it positively correlated with liver inflammation and fibrosis. Hepatic apoptotic bodies derived from apoptotic and autophagic processes can be engulfed by hepatic stellate cells (HSCs) and Kupffer cells, further stimulating the production of death receptor ligands, such as TNF-α and FasL (Marra et al., 2008). These death receptor ligands not only induce apoptosis in hepatocytes, but also activate the inflammatory signaling cascade in hepatic immune cells. TNF-α has the capacity to activate nuclear factor-kB (NF-kB) and JNK pathways by binding TNFR1, triggering a series of inflammatory responses (Marra et al., 2008). FasL stimulates the secretion of chemokines in macrophages through MyD88 signaling axis, which recruits neutrophils to the liver and potentiates inflammatory responses (Altemeier et al., 2007). In addition, hepatocyte apoptosis closely correlates with the production of transforming growth factor-β1 (TGF-β1) and the release of purinergic ligands, including UDP-glucose and UDP-fructose (Oh et al., 2012; Mederacke et al., 2022). Among which, TGF-β1 contributes to liver fibrosis through facilitating extracellular matrix deposition via TGF-β1/Smad axis in HSCs, while UDP-glucose/UDP-fructose recognizes purinergic receptor P2Y14 distributed in HSCs, resulting in the activation of HSCs and liver fibrosis (Oh et al., 2012; Mederacke et al., 2022).

### 4.2 Hepatocyte necroptosis

As indicated above, hepatocyte apoptosis is an important driver of MASH progression. But notably, in the MASH mouse model, the simultaneous inhibition of caspases at genetic and pharmacological levels can not completely block hepatocyte death, liver steatosis and inflammation, which suggests that other modalities of cell death also engage in the development of MASH (Anstee et al., 2010; Hatting et al., 2013). Under a physiological condition, the expression level of RIPK3 in hepatocytes is very low (Vucur et al., 2023). Preston et al. have demonstrated that the expression of RIPK3 was inhibited or not detected in MASH patients and human primary hepatocytes exposed to RIPK3 inducers (Preston et al., 2022). In a recent study, Vucur et al. has showed that there was a molecular switch in the hepatocyte, which could determine the specific mode of necroptosis execution and following responses (Vucur et al., 2023). NF-kB activation leads to sublethal necrosome activation in the hepatocyte and thus induces the release of DAMPs, ultimately promoting inflammation, while NF-kB inactivation prevents inflammation by accelerating necroptosis execution and limiting alarmin release (Vucur et al., 2023). Based on these studies, the low expression of RIPK3 level in hepatocytes and NF-kB activation in MASH may lead to sublethal necroptosis, which expedites the disease progression (Figure 4). Furthermore, a critical molecule in the necroptotic pathway, MLKL, can repress autophagy independent of RIPK3, and thus contributes to the liver injury induced by FFC diet (Wu et al., 2020). In turn, autophagy inhibition can increase the expression of MLKL and promote its translocation to the cell surface (Wu et al., 2020). These evidences suggest that there is an important link between MLKL expression, autophagy, and FFC diet-induced liver damage.

### 4.3 Hepatocyte pyroptosis

Recent accumulated evidence shows that hepatocyte pyroptosis contributes to the pathological progression of MASH (Figure 4). In the MASH mice induced by MCD diet, the levels of hepatic NLRP3 inflammasome and IL-1β were significantly increased, which was often concomitant with aggravated hepatic inflammation and liver injury (Vivoli et al., 2016). Another animal study also using MCD-fed mice as the MASH model, some researchers have showed that the expression levels of GSDMD and GSDMD-NT in MASH subjects were increased compared to the control, and that the knockdown of Gsdmd conferred the protection against hepatic injury induced by MCD diet (Xu et al., 2018). Similarly, in a human study, compared to MASL patients, the caspase-1 activity in the liver and serum of MASH individuals was strikingly increased and had a close association with the severity of liver damage (Gaul et al., 2021). As mentioned above, the induction of pyroptosis is accompanied by the leakage of cellular components and the release of massive inflammatory cytokines, such as IL-18 and IL-1β, directly promoting inflammatory responses. Moreover, IL-1β can also exacerbate liver inflammation by recruiting macrophages via the activation of NF-κB (Mirea et al., 2018). In addition, the released NLRP3 inflammasome particles can be engulfed by HSCs, which results in HSCs activation and α-SMA upregulation, contributing to the progression of fibrosis in MASH (Gaul et al., 2021).

### 4.4 Hepatocyte autophagy and autophagy-dependent cell death

An aberrant lipid accumulation is observed as the prerequisite for the initiation and progression of MASLD. Hepatocyte autophagy is known to contribute to the degradation of hepatic lipid droplets, which is termed lipoautophagy (Singh et al., 2009). Recently, a growing body of evidence showed the significant role of autophagy in the pathogenesis of MASLD (Gual et al., 2017; Allaire et al., 2019). In the early stage of MASLD, the level of hepatocyte autophagy is induced, which is associated with the protection against the pathological progression of the disease (Allaire et al., 2019). Hepatocyte autophagy not only blocks hepatic steatosis by facilitating the degradation of lipid droplets in hepatocytes, but also mitigates inflammatory responses and liver injury by restraining cellular oxidative stress, especially ERS and mitochondrial stress (Gual et al., 2017). Moreover, Beclin 1, a critical protein produced in autophagic process, was reported to suppress apoptosis by combing with Bcl-2 and forming a complex, thus exerting a protective role against hepatocyte damage or death (Kang et al., 2011). However, with the progression of MASLD, the autophagy activity is significantly inhibited. A recent study has observed that, in both MASH individuals and MASH murine models induced by MCD, the autophagic flux detected in liver was markedly suppressed (González-Rodríguez et al., 2014). In another report using MCD-induced MASH mouse model, enhanced autophagy suppressed liver inflammation and fibrosis, while autophagy inhibition led to the aggravation of liver injury (Figure 4) (Chen et al., 2016). Autophagy deficiency is unfavorable for the elimination of ERS and dysregulated or impaired mitochondria, thus potentiating ROS generation, which activates the inflammasome and promotes the release of inflammatory cytokines and chemokines, including TNF-α, IL-1β, as well as monocyte chemotactic protein-1 (Chen et al., 2016). Furthermore, hepatocyte autophagy inhibition also contributes to liver fibrosis (Hernández-Gea et al., 2012; Allaire et al., 2019). Despite that autophagy-dependent cell death plays an important role in mammalian embryogenesis, there is little evidence to suggest that it participates the development of MASLD.

### 4.5 Hepatocyte ferroptosis

The liver is mainly responsible for iron metabolism, and in turn, the disturbance of iron homeostasis is associated with the development of diverse liver disorders, including MASLD (Wang S. et al., 2018). As aforementioned, aberrant iron accumulation is a critical driver of ferroptosis, which has recently been confirmed to correlate with the progression of MASLD. Hepatic ferroptosis was observed in a MASH murine model induced by a MCD diet by utilizing transmission electron microscopy, and ferroptosis inhibitors strikingly ameliorated liver inflammation and fibrosis in these mice (Li et al., 2020). Moreover, a ferroptosis inducer RSL-3 exacerbates liver inflammation and fibrosis in another report using MCD-induced MASH animal model (Qi et al., 2020). The levels of several indicators of ferroptosis, such as 4-HNE and MAD, were much higher in MASH individuals than those in MASL subjects (Loguercio et al., 2001). These results provide evidence that ferroptosis engages in MASH pathogenesis and aggravates liver inflammation and fibrosis (Figure 4). Hepatocyte ferroptosis is often concomitant with the release of massive iron, which can be engulfed by macrophages and induce a series of inflammatory responses (Wang Z. et al., 2018; Pereira et al., 2019). On the one hand, macrophages can be polarized into proinflammatory types owing to excessive cellular iron in them (Pereira et al., 2019). On the other hand, iron has the capacity to modulate the production of inflammatory cytokines by regulating tricarboxylic acid cycle in macrophages (Pereira et al., 2019). It is suggested that iron contributes to cytokines production via post-transcriptional regulation, and further promotes the development of liver inflammation (Wang Z. et al., 2018). Induction of hepatocyte ferroptosis exacerbates cell death and liver injury, thus promoting liver fibrosis. Of note, autophagy has an intimate association with ferroptosis. An increased autophagic flux was observed in ferroptotic cell death and autophagy inhibition reduced ferroptosis (Liu et al., 2020). In a recent study, TMEM164 has been identified as a critical driver for autophagy-dependent ferroptosis by degrading lipid droplets, GPX4, and ferritin (Liu et al., 2023). Of note, ferroptosis has an intimate association with autophagy. An increased autophagic flux was observed in ferroptotic cell death and autophagy inhibition reduced ferroptosis (Liu et al., 2020). In a recent study, TMEM164 has been identified as a critical driver for autophagy-dependent ferroptosis by degrading lipid droplets, GPX4, and ferritin (Liu et al., 2023).

## 5 Hepatocyte signaling that regulates programmed cell death in MASH

### 5.1 YAP/TAZ

The Hippo-YAP pathway plays an important role in modulating cell growth and proliferation, thus controlling tissue homeostasis and organ size. The two downstream transcriptional coactivators, YAP and TAZ, can be regulated by the Hippo-YAP pathway and modulate the transcription of its target genes. The TAZ levels in hepatocytes were higher in MASH patients and animal models than those in the controls (Wang X. et al., 2016). Interestingly, the upregulation of TAZ was not observed in MASL subjects, which indicated that TAZ engaged in the pathological progression from MASL to MASH (Wang X. et al., 2016). The silence of hepatocyte TAZ markedly ameliorated hepatocyte death, liver inflammation and fibrosis of MASH (Wang X. et al., 2016). The Hippo-YAP pathway engages the regulation of several different forms of hepatocyte PCD, including autophagy, ferroptosis, and apoptosis. YAP/TAZ not only regulates autophagosomes degradation to control autophagic flux, but also contributes to the transition from autophagosomes into autolysosomes. Moreover, the key constituents of the Hippo-YAP pathway, STK3/STK4, play a critical role in fusing autophagosomes with lysosomes and removing intracellular cargoes. In addition, YAP/TAZ has also been confirmed to regulate a variety of processes implicated in ferroptosis by analyzing the profile of YAP/TAZ targeted-genes, including Trf1, ACSL4 and so on, which get involved in iron metabolism, oxidative responses, and lipid peroxidation. Furthermore, YAP/TAZ is considered as a potent inhibitor of apoptosis, which can restrain mitochondrial apoptotic pathway by upregulating Bcl-2 family members, and can suppress extrinsic apoptotic pathway initiated by FasL and TNF-α.

### 5.2 Notch

The Notch pathway is commonly deemed to be a cardinal player in cell differentiation, cell proliferation, and cell fate determination (Amsen et al., 2015; Bartolome et al., 2019). Notch ligands activate the corresponding receptors located on cells and cause the conformational changes in these Notch receptors, which contributes to the exposition of ADAM10 cleavage site and subsequent Notch intracellular domain (NICD) release with the assistance of γ-secretase complex (Kopan and Ilagan, 2009). The released NICD is then translocated into the nucleus in which it initiates the transcription of downstream genes, thereby exerting its multiple biological functions (Kopan and Ilagan, 2009). Under healthy conditions, the Notch pathway cannot be detected in hepatocytes, while in MASLD patients and MASLD murine models, the activity of hepatocyte Notch is obviously upregulated (Zhu et al., 2018). Moreover, the inhibition of Notch pathway strikingly ameliorated hepatic steatosis and fibrosis in the MASLD mouse model (Zhu et al., 2018). The Notch pathway engages in MASLD pathogenesis by modulating lipid metabolism, IR, oxidative stress, as well as liver inflammation and fibrosis (Zhu et al., 2018). Recently, several studies also have provided compelling evidence to support the notion that there is an intimate relationship between hepatocyte death and Notch pathway in MASLD. In FFA-treated HepG2 cells, an *in vitro* MASLD model, the Notch pathway was activated in MASLD model, and it was concomitant with hepatic steatosis and cell apoptosis, which was significantly improved by the Notch inhibitor DAPT (Zhang M. et al., 2021).After the treatment with DAPT, the autophagic flux in hepatocytes was increased, and the autophagy inhibitor chloroquine reversed the protective effects of DPAT on liver injury (Zhang M. et al., 2021). In addition, the ADSCs treatment in a MASH murine model showed enhanced liver repair and regeneration by reducing hepatocyte apoptosis via the activation of the Notch pathway (Ishida et al., 2021).

### 5.3 Hedgehog

The Hedgehog pathway is ubiquitously recognized as one of the highly conservative signaling pathways among species, which gets involved in tissue repair and regeneration, embryonic development, and tumorigenesis (Pak and Segal, 2016). The classical Hedgehog pathway mainly consists of Hedgehog, Patched, Smoothened, and Gli. The Hedgehog, as a ligand, firstly binds the cellular membrane receptor, Patched, and the effects of Hedgehog on cells depend on its concentration and exposure duration (Hirsova et al., 2013). If the Pathed is activated, it can disinhibit Smoothened, which then promotes the translocation of Gli from cytoplasm into nucleus where it has the capacity to modulate the expression of its targeted genes, thus exerting multiple functions (Hirsova et al., 2013). A plethora of studies have found that the Hedgehog pathway was aberrantly activated in both MCD-induced MASH murine models and MASH patients, and its activation had a positive correlation with the histologic severity of MASLD patients, especially liver injury and liver fibrosis (Syn et al., 2011; Guy et al., 2012). Several studies have investigated the profibrotic effects of the Hedgehog pathway in MASH, while the mechanisms of hepatocyte cell death and the Hedgehog pathway in MASH are still poorly understood. Vismodegib, a Hedgehog pathway inhibitor, attenuated liver damage partly via the blocking of apoptosis initiated by death ligands TRAIL (Hirsova et al., 2013). In addition, another Hedgehog pathway inhibitor, LDE255, was reported to reduce LC3-II production and autophagic flux, which might be a mechanism by which LDE255 inactivated HSCs induced by palmitic acids (Duan et al., 2017). Furthermore, in a recent study showed that the Hedgehog pathway participates in the regulation of hepatocyte necroptosis in the inflammation mediated by IR stress (Li et al., 2021). These observations suggest the Hedgehog pathway contributes to the progression of MASH partly through the regulation of hepatocyte cell death, but we need further investigations.

### 5.4 TAK1

TGF-β kinase 1 (TAK1) is widely considered as one of the MAP3K family members, which can be activated by multiple stimuli, such as TRAIL, IL-1, TNF-α, TGF-β, and microbial products (Wang et al., 2021). TAK1 serves as a potent activator of MAPK and NF-kB, and plays a role in regulating cell survival, immune responses, inflammatory cascades, and embryonic development (Wang et al., 2021). TAK1 can cause lipid metabolism disorder and trigger hepatic inflammation (Wang P. X. et al., 2016). Indeed, recent studies have suggested that TAK1 can be a therapeutic target for MASH, and that some pharmacological agents exerted their salutary effects on MASH via the inhibition of TAK1 signaling pathways (Liu et al., 2021; Lan et al., 2022; Liang et al., 2022), which linked TAK1 to MASH pathogenesis. Intriguingly, TAK1 also has the capacity to regulate cell death, which is a part of the mechanisms by which TAK1 contributes to MASH. NF-kB, as a downstream effector of TAK1, upregulates the expression of anti-apoptotic proteins at the transcription levels, such as IAP and c-FILP (Wang et al., 1998; Chang et al., 2006). Moreover, TAK1 reduces the production of ROS induced by TNF-α, which results in cIAP degradation, RIPK1 de-ubiquitination, and caspase-8 activation (Morioka et al., 2009). Thus, TAK1 shows protection against TNF-α-mediated apoptosis. Additionally, in steatotic hepatocytes, TAK1 also can inhibit TGF-β-induced apoptosis (Yang et al., 2014). Furthermore, TAK1 also engages in the regulation of autophagy by activating AMPK and transcriptionally regulating the expression of autophagy-associated genes. TAK1 deficiency has been confirmed to cause autophagy defect, which results in liver steatosis and hepatic tumorigenesis (Herrero-Martín et al., 2009; Inokuchi-Shimizu et al., 2014). However, TAK1 potentiates the execution of necroptotic pathway. TAK1 contributes to the activation of RIPK1 and RIPK3, which in turn activates TAK1, thus forming TAK1-RIPK1-RIPK3 loop and ultimately leading to necroptosis (Morioka et al., 2014). TAK1 also has the capacity to switch cells fate from apoptosis to necroptosis. Taken together, TAK1 is multifactorial and contributes to both cellular protection and death.

### 5.5 AMPK

Adenosine monophosphate (AMP)-activated protein kinase (AMPK) is extensively deemed to be an important metabolic modulator and energy sensor (Hardie, 2011). AMPK senses the cell energy status and its activity mainly regulated by ATP and AMP (Hardie, 2011). Specifically, AMP allosterically activates AMPK while ATP represses it. In order to maintain energy homeostasis, AMPK regulates catabolism processes, like FA β-oxidation, and anabolic processes, like the synthesis of three major nutrient substance (Guicciardi et al., 2020). The activity of AMPK has been reported to decline in MASH, and the specific knockout of hepatic AMPK correlated with the exacerbation of liver damage, suggesting the significant role of decreased AMPK in MASH pathogenesis (Smith et al., 2016; Zhao et al., 2020). Besides the effects of AMPK exert on energy and metabolism regulation, AMPK also participates in regulating cell death. As indicated above, activated caspases-6 by caspase-3/7 promotes the cleavage of t-Bid and the release of CtyC, which in turn promotes the activation of caspase-3/7, leading to a feed-forward loop formation and excessive apoptosis (Zhao et al., 2020). AMPK was found to dampen caspase-6 activation and thus reduced hepatocyte apoptosis, which ultimately ameliorated liver injury in MASH (Zhao et al., 2020). Moreover, AMPK can catalyze the phosphorylation of mTOR and inactivate it, further inducing autophagy, as mTOR has a negative regulation for autophagy (Kim and Guan, 2015; Madiraju et al., 2016). Furthermore, AMPK also engages in ferroptosis regulation. AMPK is able to phosphorylate Beclin 1, which inhibits the system Xc-by binding SCL7A11, and ultimately promotes ferroptosis (Song et al., 2018). However, whether AMPK regulates hepatocyte ferroptosis in MASH still remains elusive, which warrants more in-depth investigations. Taken together, reduced AMPK activity in MASH exacerbates liver injury by promoting hepatocyte apoptosis and blocking hepatocyte autophagy, which could propose a novel therapeutic strategy for MASH.

## 6 Resolution of hepatocyte PCD in MASH

Up to now, the lifestyle interventions including exercise control and dietary modifications are the only managements for MASLD. However, single altering lifestyles is insufficient to improve NASH completely. Recently, an increasing body of studies have suggested that hepatocyte PCD played an essential role in MASH pathogenesis, and on top of autophagy, other forms of hepatocyte PCD, including apoptosis, necroptosis, pyroptosis, and ferroptosis, all greatly contributed to the transition from MASL to MASH. Therefore, resolving hepatocyte PCD can be a candidate therapeutic avenue for MASH. Indeed, several pharmacological agents targeting one or more forms of PCD have been confirmed to improve MASH in many animal studies (Table 1). As aforementioned, the caspase family plays an important role in apoptosis execution. The caspase inhibitors, such as Emricasan, VX-166, and GS-9450, were reported to block hepatocyte apoptosis and ameliorate hepatic inflammation and fibrosis in MASH animal models induced by HFD or MCD (Witek et al., 2009; Anstee et al., 2010; Barreyro et al., 2015). However, in recent clinical trials, Emricasan could not ameliorate liver inflammation and fibrosis in MASH patients (Harrison et al., 2020; Frenette et al., 2021). RIPA-56, an inhibitor of RIPK1, which is recognized as the gatekeeper of necroptotic pathway, mitigated hepatic inflammation and fibrosis in the HFD-induced MASH murine model by blocking necroptosis (Majdi et al., 2020). RIPK3 is also a critical regulator in necroptosis and has the capacity to trigger necroptosis independent of RIPK1. In a choline-deficient HFD-induced MASH murine model, RIPK3 inhibition aggravated IR and hepatic steatosis (Gautheron et al., 2016). Herein, it is highly necessary to develop the specific RIPK3 inhibitors which only take hepatocytes as target. NLRP3 acts as a cardinal mediator in the execution of pyroptosis, and inhibiting NLRP3 is emerging as an effective method to block pyroptosis. Liraglutide, a GLP-1 analogue, ameliorated MASH in an *in vitro* model utilizing PA/LPS treated hepatocytes partly through restraining NLRP3 inflammasome and pyroptotic pathway (Yu et al., 2019). Fer1, as one kind of ferroptosis inhibitor, exhibited protective effects against liver inflammation and damage in MCD-induced MASH animal models via the inhibition of hepatocyte ferroptosis (Li et al., 2020; Yang et al., 2020; Zhu et al., 2021; Jiang J. J. et al., 2022). Herein, developing cell targeted pharmacological agents which exert its function by regulating ferroptosis is of great benefit to more accurate treatment for MASH. Moreover, some FDA-approved drugs ranging from exenatide to metformin, TFEB inducers, and rapamycin contributed to the improvement of MASH partly by inducing autophagy in different animal models (Chen et al., 2016; Kim et al., 2017; Lim et al., 2018; Li et al., 2019). But notably, these drugs also improved MASH by other mechanisms.

**TABLE 1:** Potential therapeutic pharmacological agents for MASH targeting PCD.

Pharmacological agents	Experimental model	Impact on PCD	Therapeutic effects	Ref
RIPA-56	HFD-induced MASH murine model	Necroptosis inhibition	Ameliorates liver inflammation and fibrosis	Majdi et al. (2020)
Liraglutide	Human HepG2 cells exposed to PA and LPS	Pyroptosis inhibition	Slows the progression of MASH	Yu et al. (2019)
Fer1	MCD-induced MASH murine model	Ferroptosis inhibition	Ameliorates liver inflammation and fibrosis	Li et al. (2020)
Tectorigenin	HFD-induced MASH murine model and human HepG2 cells exposed to free fatty acids	Autophagy induction and pyroptosis inhibition	Ameliorates liver inflammation and injury	Zhu et al. (2022)

Abbreviations: MASH, metabolic dysfunction-associated steatohepatitis; PCD, programmed cell death; HFD, high fat diet; MCD, methionine-choline deficient diet; PA, palmitic; LPS, lipopolysaccharides.

Besides the aforementioned pharmacological agents only targeting single PCD, there still exist many effective therapeutic agents which simultaneously target two or more modalities of PCD. Tectorigenin, as one of primary monomers of blueberry, strikingly attenuated liver inflammation and damage in both *in vitro* and *in vivo* MASH models through inducing autophagy and blocking pyroptosis (Zhu et al., 2022).

Taken together, regulating the activity of hepatocyte PCD is emerging as a potential effective therapy for MASH treatment. However, the therapeutic effects of the agents targeting hepatocyte PCD in MASH have just been evaluated in preclinical studies, which warrants further investigation in clinical practice.

## 7 Conclusion

MASH greatly threatens human health owing to its consecutively increased incidence and prevalence on a globe scale (Younossi et al., 2018). Single lifestyle intervention is insufficient to improve NASH completely (Diehl et al., 2019). Explicitly understanding the pathogenesis of MASH and finding some novel effective therapeutic points of intervention are the most urgent tasks for MASH management. In recent studies, hepatocyte PCD, including apoptosis, necroptosis, pyroptosis, autophagy, and ferroptosis, have attracted much attention as a momentous partaker in MASH pathogenesis (Feldstein et al., 2003; Nan et al., 2007; Allaire et al., 2019; Gautheron et al., 2020). Metabolic disorders in MASLD, such as lipotoxicity, glucotoxicity, iron overload, copper deficiency, and PAMPs/DAMPs, trigger different modalities of hepatocyte PCD, which serve as potent drivers of the pathological progression from MASL to MASH mainly through facilitating liver inflammation and fibrosis (Feldstein et al., 2003; Nan et al., 2007; Volkmann et al., 2007). Indeed, several potential therapeutic pharmacological agents for MASH have been confirmed to exert their salutary effects partly by regulating the activity of one or more type of hepatocyte PCD (Ezquerro et al., 2019; Nasiri-Ansari et al., 2021; Shen et al., 2021; Zhu et al., 2022). Herein, hepatocyte PCD seems to be a new underlying therapeutic target for MASH.

Although targeting hepatocyte PCD sheds light on MASH treatment, it still remains a tremendous challenge to develop safe and effective drugs utilized in MASH individuals which take hepatocyte PCD as target. As aforementioned, multiple modalities of hepatocyte PCD are implicated in MASH pathogenesis, and each form of PCD has an interaction with other types of cell death. The intricate interaction among different modalities of hepatocyte PCD is yet to be thoroughly understood. Moreover, although some pharmacological agents have been proved to improve MASH partly by regulating the activity of hepatocyte PCD, they also ameliorate MASH via other mechanisms. The number of potential therapeutic drugs for MASH only targeting PCD remains scarce. Additionally, besides hepatocytes, PCD can also occur in other cell types in liver, including macrophages and HSCs. It is noteworthy that PCD in different cell types exerts different effects. Thus, great efforts need to be put in the development of drug-targeted delivery system. Taken together, more attention should be paid to gain further insights into the intricate interaction among different modalities of hepatocyte PCD and to develop more safe and effective drugs which improve MASH by specifically targeting hepatocyte PCD.

## Figures and Tables

**Table:** 

**NAFLD**	Nonalcoholic Liver Disease
**NAFL**	Nonalcoholic Fatty Liver
**NASH**	Nonalcoholic Steatohepatitis
**HCC**	Hepatocellular Carcinoma
**MASLD**	Metabolic Dysfunction-Associated Steatotic Liver Disease
**MASL**	Metabolic Dysfunction-Associated Steatotic Liver
**MASH**	Metabolic Dysfunction-Associated Steatohepatitis
**PCD**	Programmed Cell Death
**TNF**	Tumor Necrosis Factor
**RIPK1**	Receptor-Interacting Protein Kinase-1
**Bcl-2**	B-cell Lymphoma-2
**MOMP**	Mitochondrial Outer Membrane Pore Formation
**BID**	BH3-Interacting Domain Death Agonist
**CytC**	Cytochrome c
**ERS**	Endoplasmic Reticulum Stress
**DAMPs**	Damaged Associated Molecular Patterns
**MLKL**	Pseudokinase Mixed Lineage Domain-Like
**PAMPs**	Pathogen-Associated Molecular Patterns
**NLRs**	Nod-Like Receptors
**AIM2**	Absent In Melanoma-2
**GSDMD**	Gasdermin D
**IL**	Interleukin
**LPS**	Lipopolysaccharide
**CMA**	Chaperone-Mediated Autophagy
**PUFAs**	Polyunsaturated Fatty Acids
**LPCAT3**	Lysophosphatidylcholine Acyltransferase 3
**ACSL4**	Acyl-CoA Synthetase Long-Chain Family Member 4
**FFAs**	Free Fatty Acids
**TGs**	Triglycerides
**LPC**	Lysophosphatidylcholine
**FC**	Free Cholesterol
**HFD**	High Fat Diet
**IR**	Insulin Resistance
**MAMPs**	Microbe-Associated Molecular Patterns
**MCD**	Methionine-Choline Deficient
**HSCs**	Hepatic Stellate Cells
**NF-kB**	Nuclear Factor-kB
**TGF-β1**	Transforming Growth Factor-β1
**NICD**	Notch Intracellular Domain
**TAK1**	TGF-β kinase 1
**AMP**	Adenosine Monophosphate
**AMPK**	AMP-Activated Protein Kinase

## References

[B1] Aigner E. Strasser M. Haufe H. Sonnweber T. Hohla F. Stadlmayr A. (2010). A role for low hepatic copper concentrations in nonalcoholic Fatty liver disease. Am. J. Gastroenterol. 105 (9), 1978–1985. 10.1038/ajg.2010.170 20407430

[B2] Aigner E. Theurl I. Haufe H. Seifert M. Hohla F. Scharinger L. (2008). Copper availability contributes to iron perturbations in human nonalcoholic fatty liver disease. Gastroenterology 135 (2), 680–688. 10.1053/j.gastro.2008.04.007 18505688

[B3] Aigner E. Weiss G. Datz C. (2015). Dysregulation of iron and copper homeostasis in nonalcoholic fatty liver. World J. Hepatol. 7 (2), 177–188. 10.4254/wjh.v7.i2.177 25729473 PMC4342600

[B4] Akazawa Y. Cazanave S. Mott J. L. Elmi N. Bronk S. F. Kohno S. (2010). Palmitoleate attenuates palmitate-induced Bim and PUMA up-regulation and hepatocyte lipoapoptosis. J. Hepatol. 52 (4), 586–593. 10.1016/j.jhep.2010.01.003 20206402 PMC2847010

[B5] Alkhouri N. Carter-Kent C. Feldstein A. E. (2011). Apoptosis in nonalcoholic fatty liver disease: diagnostic and therapeutic implications. Expert Rev. Gastroenterol. Hepatol. 5 (2), 201–212. 10.1586/egh.11.6 21476915 PMC3119461

[B6] Allaire M. Rautou P. E. Codogno P. Lotersztajn S. (2019). Autophagy in liver diseases: time for translation? J. Hepatol. 70 (5), 985–998. 10.1016/j.jhep.2019.01.026 30711404

[B7] Altemeier W. A. Zhu X. Berrington W. R. Harlan J. M. Liles W. C. (2007). Fas (CD95) induces macrophage proinflammatory chemokine production via a MyD88-dependent, caspase-independent pathway. J. Leukoc. Biol. 82 (3), 721–728. 10.1189/jlb.1006652 17576821 PMC4492281

[B8] Amarante-Mendes G. P. Adjemian S. Branco L. M. Zanetti L. C. Weinlich R. Bortoluci K. R. (2018). Pattern recognition receptors and the host cell death molecular machinery. Front. Immunol. 9, 2379. 10.3389/fimmu.2018.02379 30459758 PMC6232773

[B9] Amsen D. Helbig C. Backer R. A. (2015). Notch in T Cell differentiation: all things considered. Trends Immunol. 36 (12), 802–814. 10.1016/j.it.2015.10.007 26617322

[B10] Anstee Q. M. Concas D. Kudo H. Levene A. Pollard J. Charlton P. (2010). Impact of pan-caspase inhibition in animal models of established steatosis and non-alcoholic steatohepatitis. J. Hepatol. 53 (3), 542–550. 10.1016/j.jhep.2010.03.016 20557969

[B11] Arora A. S. Jones B. J. Patel T. C. Bronk S. F. Gores G. J. (1997). Ceramide induces hepatocyte cell death through disruption of mitochondrial function in the rat. Hepatology 25 (4), 958–963. 10.1002/hep.510250428 9096604

[B12] Baehrecke E. H. (2003). Autophagic programmed cell death in Drosophila. Cell Death Differ. 10 (9), 940–945. 10.1038/sj.cdd.4401280 12934068

[B13] Bani-Hani A. H. Leslie J. A. Asanuma H. Dinarello C. A. Campbell M. T. Meldrum D. R. (2009). IL-18 neutralization ameliorates obstruction-induced epithelial-mesenchymal transition and renal fibrosis. Kidney Int. 76 (5), 500–511. 10.1038/ki.2009.216 19536084

[B14] Barreyro F. J. Holod S. Finocchietto P. V. Camino A. M. Aquino J. B. Avagnina A. (2015). The pan-caspase inhibitor Emricasan (IDN-6556) decreases liver injury and fibrosis in a murine model of non-alcoholic steatohepatitis. Liver Int. 35 (3), 953–966. 10.1111/liv.12570 24750664

[B15] Barreyro F. J. Kobayashi S. Bronk S. F. Werneburg N. W. Malhi H. Gores G. J. (2007). Transcriptional regulation of Bim by FoxO3A mediates hepatocyte lipoapoptosis. J. Biol. Chem. 282 (37), 27141–27154. 10.1074/jbc.M704391200 17626006

[B16] Bartolome A. Zhu C. Sussel L. Pajvani U. B. (2019). Notch signaling dynamically regulates adult β cell proliferation and maturity. J. Clin. Invest. 129 (1), 268–280. 10.1172/JCI98098 30375986 PMC6307965

[B17] Bedoui S. Herold M. J. Strasser A. (2020). Emerging connectivity of programmed cell death pathways and its physiological implications. Nat. Rev. Mol. Cell Biol. 21 (11), 678–695. 10.1038/s41580-020-0270-8 32873928

[B18] Bernales S. Schuck S. Walter P. (2007). ER-phagy: selective autophagy of the endoplasmic reticulum. Autophagy 3 (3), 285–287. 10.4161/auto.3930 17351330

[B19] Berson E. L. Rosner B. Sandberg M. A. Weigel-DiFranco C. Moser A. Brockhurst R. J. (2004). Further evaluation of docosahexaenoic acid in patients with retinitis pigmentosa receiving vitamin A treatment: subgroup analyses. Arch. Ophthalmol. 122 (9), 1306–1314. 10.1001/archopht.122.9.1306 15364709

[B20] Brault M. Olsen T. M. Martinez J. Stetson D. B. Oberst A. (2018). Intracellular nucleic acid sensing triggers necroptosis through synergistic type I IFN and TNF signaling. J. Immunol. 200 (8), 2748–2756. 10.4049/jimmunol.1701492 29540580 PMC5893403

[B21] Buzun K. Gornowicz A. Lesyk R. Bielawski K. Bielawska A. (2021). Autophagy modulators in cancer therapy. Int. J. Mol. Sci. 22 (11), 5804. 10.3390/ijms22115804 34071600 PMC8199315

[B22] Calzadilla Bertot L. Adams L. A. (2016). The natural course of non-alcoholic fatty liver disease. Int. J. Mol. Sci. 17 (5), 774. 10.3390/ijms17050774 27213358 PMC4881593

[B23] Cazanave S. C. Elmi N. A. Akazawa Y. Bronk S. F. Mott J. L. Gores G. J. (2010). CHOP and AP-1 cooperatively mediate PUMA expression during lipoapoptosis. Am. J. Physiol. Gastrointest. Liver Physiol. 299 (1), G236–G243. 10.1152/ajpgi.00091.2010 20430872 PMC2904106

[B24] Cazanave S. C. Mott J. L. Bronk S. F. Werneburg N. W. Fingas C. D. Meng X. W. (2011). Death receptor 5 signaling promotes hepatocyte lipoapoptosis. J. Biol. Chem. 286 (45), 39336–39348. 10.1074/jbc.M111.280420 21941003 PMC3234758

[B25] Chang L. Kamata H. Solinas G. Luo J. L. Maeda S. Venuprasad K. (2006). The E3 ubiquitin ligase itch couples JNK activation to TNFalpha-induced cell death by inducing c-FLIP(L) turnover. Cell 124 (3), 601–613. 10.1016/j.cell.2006.01.021 16469705

[B26] Chen Q. Shi P. Wang Y. Zou D. Wu X. Wang D. (2019). GSDMB promotes non-canonical pyroptosis by enhancing caspase-4 activity. J. Mol. Cell Biol. 11 (6), 496–508. 10.1093/jmcb/mjy056 30321352 PMC6734491

[B27] Chen R. Wang Q. Song S. Liu F. He B. Gao X. (2016). Protective role of autophagy in methionine-choline deficient diet-induced advanced nonalcoholic steatohepatitis in mice. Eur. J. Pharmacol. 770, 126–133. 10.1016/j.ejphar.2015.11.012 26593434

[B28] Cifone M. G. De Maria R. Roncaioli P. Rippo M. R. Azuma M. Lanier L. L. (1994). Apoptotic signaling through CD95 (Fas/Apo-1) activates an acidic sphingomyelinase. J. Exp. Med. 180 (4), 1547–1552. 10.1084/jem.180.4.1547 7523573 PMC2191710

[B29] Cox C. L. Stanhope K. L. Schwarz J. M. Graham J. L. Hatcher B. Griffen S. C. (2012). Consumption of fructose- but not glucose-sweetened beverages for 10 weeks increases circulating concentrations of uric acid, retinol binding protein-4, and gamma-glutamyl transferase activity in overweight/obese humans. Nutr. Metab. (Lond) 9 (1), 68. 10.1186/1743-7075-9-68 22828276 PMC3463498

[B30] Cuervo A. M. Dice J. F. (1996). A receptor for the selective uptake and degradation of proteins by lysosomes. Science 273 (5274), 501–503. 10.1126/science.273.5274.501 8662539

[B31] Czaja M. J. (2010). Autophagy in health and disease. 2. Regulation of lipid metabolism and storage by autophagy: pathophysiological implications. Am. J. Physiol. Cell Physiol. 298 (5), C973–C978. 10.1152/ajpcell.00527.2009 20089934 PMC2867392

[B32] de Vasconcelos N. M. Van Opdenbosch N. Van Gorp H. Parthoens E. Lamkanfi M. (2019). Single-cell analysis of pyroptosis dynamics reveals conserved GSDMD-mediated subcellular events that precede plasma membrane rupture. Cell Death Differ. 26 (1), 146–161. 10.1038/s41418-018-0106-7 29666477 PMC6294780

[B33] Dhuriya Y. K. Sharma D. (2018). Necroptosis: a regulated inflammatory mode of cell death. J. Neuroinflammation 15 (1), 199. 10.1186/s12974-018-1235-0 29980212 PMC6035417

[B34] Diehl A. M. Farpour-Lambert N. J. Zhao L. Tilg H. (2019). Why we need to curb the emerging worldwide epidemic of nonalcoholic fatty liver disease. Nat. Metab. 1 (11), 1027–1029. 10.1038/s42255-019-0140-x 32694863

[B35] Dikic I. Elazar Z. (2018). Mechanism and medical implications of mammalian autophagy. Nat. Rev. Mol. Cell Biol. 19 (6), 349–364. 10.1038/s41580-018-0003-4 29618831

[B36] Ding W. X. Li M. Chen X. Ni H. M. Lin C. W. Gao W. (2010). Autophagy reduces acute ethanol-induced hepatotoxicity and steatosis in mice. Gastroenterology 139 (5), 1740–1752. 10.1053/j.gastro.2010.07.041 20659474 PMC4129642

[B37] Dixon S. J. Lemberg K. M. Lamprecht M. R. Skouta R. Zaitsev E. M. Gleason C. E. (2012). Ferroptosis: an iron-dependent form of nonapoptotic cell death. Cell 149 (5), 1060–1072. 10.1016/j.cell.2012.03.042 22632970 PMC3367386

[B38] Duan N. N. Liu X. J. Wu J. (2017). Palmitic acid elicits hepatic stellate cell activation through inflammasomes and hedgehog signaling. Life Sci. 176, 42–53. 10.1016/j.lfs.2017.03.012 28322865

[B39] Duewell P. Kono H. Rayner K. J. Sirois C. M. Vladimer G. Bauernfeind F. G. (2010). NLRP3 inflammasomes are required for atherogenesis and activated by cholesterol crystals. Nature 464 (7293), 1357–1361. 10.1038/nature08938 20428172 PMC2946640

[B40] Ezquerro S. Mocha F. Frühbeck G. Guzmán-Ruiz R. Valentí V. Mugueta C. (2019). Ghrelin reduces TNF-α-induced human hepatocyte apoptosis, autophagy, and pyroptosis: role in obesity-associated NAFLD. J. Clin. Endocrinol. Metab. 104 (1), 21–37. 10.1210/jc.2018-01171 30137403

[B41] Fan J. G. Li X. Y. (2023). NAFLD renaming to MAFLD, MASLD: background, similarities, differences, and countermeasures. Zhonghua Gan Zang Bing Za Zhi 31 (8), 789–792. 10.3760/cma.j.cn501113-20230809-00042 37723058 PMC12814010

[B42] Feldstein A. E. Canbay A. Angulo P. Taniai M. Burgart L. J. Lindor K. D. (2003). Hepatocyte apoptosis and fas expression are prominent features of human nonalcoholic steatohepatitis. Gastroenterology 125 (2), 437–443. 10.1016/s0016-5085(03)00907-7 12891546

[B43] Filali-Mouncef Y. Hunter C. Roccio F. Zagkou S. Dupont N. Primard C. (2022). The ménage à trois of autophagy, lipid droplets and liver disease. Autophagy 18 (1), 50–72. 10.1080/15548627.2021.1895658 33794741 PMC8865253

[B44] Frenette C. Kayali Z. Mena E. Mantry P. S. Lucas K. J. Neff G. (2021). Emricasan to prevent new decompensation in patients with NASH-related decompensated cirrhosis. J. hepatology 74 (2), 274–282. 10.1016/j.jhep.2020.09.029 33038432

[B45] Fuchs Y. Steller H. (2011). Programmed cell death in animal development and disease. Cell 147 (4), 742–758. 10.1016/j.cell.2011.10.033 22078876 PMC4511103

[B46] Galluzzi L. Vitale I. Aaronson S. A. Abrams J. M. Adam D. Agostinis P. (2018). Molecular mechanisms of cell death: recommendations of the nomenclature committee on cell death 2018. Cell Death Differ. 25 (3), 486–541. 10.1038/s41418-017-0012-4 29362479 PMC5864239

[B47] García-Ruiz C. Colell A. Marí M. Morales A. Fernández-Checa J. C. (1997). Direct effect of ceramide on the mitochondrial electron transport chain leads to generation of reactive oxygen species. Role of mitochondrial glutathione. J. Biol. Chem. 272 (17), 11369–11377. 10.1074/jbc.272.17.11369 9111045

[B48] Gaul S. Leszczynska A. Alegre F. Kaufmann B. Johnson C. D. Adams L. A. (2021). Hepatocyte pyroptosis and release of inflammasome particles induce stellate cell activation and liver fibrosis. J. Hepatol. 74 (1), 156–167. 10.1016/j.jhep.2020.07.041 32763266 PMC7749849

[B49] Gautheron J. Gores G. J. Rodrigues C. M. P. (2020). Lytic cell death in metabolic liver disease. J. Hepatol. 73 (2), 394–408. 10.1016/j.jhep.2020.04.001 32298766 PMC7371520

[B50] Gautheron J. Vucur M. Schneider A. T. Severi I. Roderburg C. Roy S. (2016). The necroptosis-inducing kinase RIPK3 dampens adipose tissue inflammation and glucose intolerance. Nat. Commun. 7, 11869. 10.1038/ncomms11869 27323669 PMC4919522

[B51] González-Rodríguez A. Mayoral R. Agra N. Valdecantos M. P. Pardo V. Miquilena-Colina M. E. (2014). Impaired autophagic flux is associated with increased endoplasmic reticulum stress during the development of NAFLD. Cell Death Dis. 5 (4), e1179. 10.1038/cddis.2014.162 24743734 PMC4001315

[B52] Green D. R. (2022). The future of death. Cold Spring Harb. Perspect. Biol. 14 (4), a041111. 10.1101/cshperspect.a041111 36456104 PMC9732899

[B53] Griffioen A. W. Nowak-Sliwinska P. (2022). Programmed cell death lives. Apoptosis 27 (9-10), 619–621. 10.1007/s10495-022-01758-5 35943678 PMC9361233

[B54] Gual P. Gilgenkrantz H. Lotersztajn S. (2017). Autophagy in chronic liver diseases: the two faces of Janus. Am. J. Physiol. Cell Physiol. 312 (3), C263–c273. 10.1152/ajpcell.00295.2016 27903585

[B55] Guicciardi M. E. Nakao Y. Gores G. J. (2020). The metabolic sensor adenosine monophosphate-activated protein kinase regulates apoptosis in nonalcoholic steatohepatitis. Hepatology 72 (3), 1139–1141. 10.1002/hep.31294 32342535

[B56] Guy C. D. Suzuki A. Zdanowicz M. Abdelmalek M. F. Burchette J. Unalp A. (2012). Hedgehog pathway activation parallels histologic severity of injury and fibrosis in human nonalcoholic fatty liver disease. Hepatology 55 (6), 1711–1721. 10.1002/hep.25559 22213086 PMC3499103

[B57] Haeggström J. Z. Funk C. D. (2011). Lipoxygenase and leukotriene pathways: biochemistry, biology, and roles in disease. Chem. Rev. 111 (10), 5866–5898. 10.1021/cr200246d 21936577

[B58] Han J. Zhong C. Q. Zhang D. W. (2011). Programmed necrosis: backup to and competitor with apoptosis in the immune system. Nat. Immunol. 12 (12), 1143–1149. 10.1038/ni.2159 22089220

[B59] Hardie D. G. (2011). AMP-activated protein kinase: an energy sensor that regulates all aspects of cell function. Genes Dev. 25 (18), 1895–1908. 10.1101/gad.17420111 21937710 PMC3185962

[B60] Harrison S. A. Goodman Z. Jabbar A. Vemulapalli R. Younes Z. H. Freilich B. (2020). A randomized, placebo-controlled trial of emricasan in patients with N ASH and F1-F3 fibrosis. J. hepatology 72 (5), 816–827. 10.1016/j.jhep.2019.11.024 31887369

[B61] Hatting M. Zhao G. Schumacher F. Sellge G. Al Masaoudi M. Gaβler N. (2013). Hepatocyte caspase-8 is an essential modulator of steatohepatitis in rodents. Hepatology 57 (6), 2189–2201. 10.1002/hep.26271 23339067

[B62] Hernández-Gea V. Ghiassi-Nejad Z. Rozenfeld R. Gordon R. Fiel M. I. Yue Z. (2012). Autophagy releases lipid that promotes fibrogenesis by activated hepatic stellate cells in mice and in human tissues. Gastroenterology 142 (4), 938–946. 10.1053/j.gastro.2011.12.044 22240484 PMC3439519

[B63] Herrero-Martín G. Høyer-Hansen M. García-García C. Fumarola C. Farkas T. López-Rivas A. (2009). TAK1 activates AMPK-dependent cytoprotective autophagy in TRAIL-treated epithelial cells. EMBO J. 28 (6), 677–685. 10.1038/emboj.2009.8 19197243 PMC2666037

[B64] Hirsova P. Ibrahim S. H. Bronk S. F. Yagita H. Gores G. J. (2013). Vismodegib suppresses TRAIL-mediated liver injury in a mouse model of nonalcoholic steatohepatitis. PLoS One 8 (7), e70599. 10.1371/journal.pone.0070599 23894677 PMC3718793

[B65] Inokuchi-Shimizu S. Park E. J. Roh Y. S. Yang L. Zhang B. Song J. (2014). TAK1-mediated autophagy and fatty acid oxidation prevent hepatosteatosis and tumorigenesis. J. Clin. Invest. 124 (8), 3566–3578. 10.1172/JCI74068 24983318 PMC4109552

[B66] Iorga A. Dara L. Kaplowitz N. (2017). Drug-induced liver injury: cascade of events leading to cell death, apoptosis or necrosis. Int. J. Mol. Sci. 18 (5), 1018. 10.3390/ijms18051018 28486401 PMC5454931

[B67] Ishida K. Seki A. Kawaguchi K. Nasti A. Yamato M. Inui H. (2021). Restorative effect of adipose tissue-derived stem cells on impaired hepatocytes through Notch signaling in non-alcoholic steatohepatitis mice. Stem Cell Res. 54, 102425. 10.1016/j.scr.2021.102425 34119957

[B68] Jaiswal N. Maurya C. K. Arha D. Avisetti D. R. Prathapan A. Raj P. S. (2015). Fructose induces mitochondrial dysfunction and triggers apoptosis in skeletal muscle cells by provoking oxidative stress. Apoptosis 20 (7), 930–947. 10.1007/s10495-015-1128-y 25913123

[B69] Jensen T. Abdelmalek M. F. Sullivan S. Nadeau K. J. Green M. Roncal C. (2018). Fructose and sugar: a major mediator of non-alcoholic fatty liver disease. J. Hepatol. 68 (5), 1063–1075. 10.1016/j.jhep.2018.01.019 29408694 PMC5893377

[B70] Jia M. Zhang H. Qin Q. Hou Y. Zhang X. Chen D. (2021). Ferroptosis as a new therapeutic opportunity for nonviral liver disease. Eur. J. Pharmacol. 908, 174319. 10.1016/j.ejphar.2021.174319 34252441

[B71] Jiang J. J. Zhang G. F. Zheng J. Y. Sun J. H. Ding S. B. (2022b). Targeting mitochondrial ROS-mediated ferroptosis by quercetin alleviates high-fat diet-induced hepatic lipotoxicity. Front. Pharmacol. 13, 876550. 10.3389/fphar.2022.876550 35496312 PMC9039018

[B72] Jiang P. Mizushima N. (2014). Autophagy and human diseases. Cell Res. 24 (1), 69–79. 10.1038/cr.2013.161 24323045 PMC3879707

[B73] Jiang Y. Huo Z. Qi X. Zuo T. Wu Z. (2022a). Copper-induced tumor cell death mechanisms and antitumor theragnostic applications of copper complexes. Nanomedicine (Lond) 17 (5), 303–324. 10.2217/nnm-2021-0374 35060391

[B74] Johnson R. J. Rivard C. Lanaspa M. A. Otabachian-Smith S. Ishimoto T. Cicerchi C. (2013). Fructokinase, fructans, intestinal permeability, and metabolic syndrome: an equine connection? J. Equine Vet. Sci. 33 (2), 120–126. 10.1016/j.jevs.2012.05.004 23439477 PMC3576823

[B75] Kagan V. E. Mao G. Qu F. Angeli J. P. Doll S. Croix C. S. (2017). Oxidized arachidonic and adrenic PEs navigate cells to ferroptosis. Nat. Chem. Biol. 13 (1), 81–90. 10.1038/nchembio.2238 27842066 PMC5506843

[B76] Kambara H. Liu F. Zhang X. Liu P. Bajrami B. Teng Y. (2018). Gasdermin D exerts anti-inflammatory effects by promoting neutrophil death. Cell Rep. 22 (11), 2924–2936. 10.1016/j.celrep.2018.02.067 29539421 PMC5878047

[B77] Kang R. Zeh H. J. Lotze M. T. Tang D. (2011). The Beclin 1 network regulates autophagy and apoptosis. Cell Death Differ. 18 (4), 571–580. 10.1038/cdd.2010.191 21311563 PMC3131912

[B78] Kang Z. Qiao N. Liu G. Chen H. Tang Z. Li Y. (2019). Copper-induced apoptosis and autophagy through oxidative stress-mediated mitochondrial dysfunction in male germ cells. Toxicol Vitro 61, 104639. 10.1016/j.tiv.2019.104639 31491480

[B79] Kast D. J. Dominguez R. (2017). The cytoskeleton-autophagy connection. Curr. Biol. 27 (8), R318–r326. 10.1016/j.cub.2017.02.061 28441569 PMC5444402

[B80] Kaushik S. Cuervo A. M. (2012). Chaperone-mediated autophagy: a unique way to enter the lysosome world. Trends Cell Biol. 22 (8), 407–417. 10.1016/j.tcb.2012.05.006 22748206 PMC3408550

[B81] Kaushik S. Cuervo A. M. (2018). The coming of age of chaperone-mediated autophagy. Nat. Rev. Mol. Cell Biol. 19 (6), 365–381. 10.1038/s41580-018-0001-6 29626215 PMC6399518

[B82] Kerr J. F. Wyllie A. H. Currie A. R. (1972). Apoptosis: a basic biological phenomenon with wide-ranging implications in tissue kinetics. Br. J. Cancer 26 (4), 239–257. 10.1038/bjc.1972.33 4561027 PMC2008650

[B83] Kim S. H. Kim G. Han D. H. Lee M. Kim I. Kim B. (2017). Ezetimibe ameliorates steatohepatitis via AMP activated protein kinase-TFEB-mediated activation of autophagy and NLRP3 inflammasome inhibition. Autophagy 13 (10), 1767–1781. 10.1080/15548627.2017.1356977 28933629 PMC5640190

[B84] Kim Y. C. Guan K. L. (2015). mTOR: a pharmacologic target for autophagy regulation. J. Clin. Invest. 125 (1), 25–32. 10.1172/JCI73939 25654547 PMC4382265

[B85] Kopan R. Ilagan M. X. (2009). The canonical Notch signaling pathway: unfolding the activation mechanism. Cell 137 (2), 216–233. 10.1016/j.cell.2009.03.045 19379690 PMC2827930

[B86] Koyama Y. Brenner D. A. (2017). Liver inflammation and fibrosis. J. Clin. Invest. 127 (1), 55–64. 10.1172/JCI88881 28045404 PMC5199698

[B87] Kroemer G. Levine B. (2008). Autophagic cell death: the story of a misnomer. Nat. Rev. Mol. Cell Biol. 9 (12), 1004–1010. 10.1038/nrm2529 18971948 PMC2727358

[B88] Lan T. Jiang S. Zhang J. Weng Q. Yu Y. Li H. (2022). Breviscapine alleviates NASH by inhibiting TGF-β-activated kinase 1-dependent signaling. Hepatology 76 (1), 155–171. 10.1002/hep.32221 34717002 PMC9299589

[B89] Lanaspa M. A. Sanchez-Lozada L. G. Choi Y. J. Cicerchi C. Kanbay M. Roncal-Jimenez C. A. (2012). Uric acid induces hepatic steatosis by generation of mitochondrial oxidative stress: potential role in fructose-dependent and -independent fatty liver. J. Biol. Chem. 287 (48), 40732–40744. 10.1074/jbc.M112.399899 23035112 PMC3504786

[B90] Li C. Sheng M. Lin Y. Xu D. Tian Y. Zhan Y. (2021). Functional crosstalk between myeloid Foxo1-β-catenin axis and Hedgehog/Gli1 signaling in oxidative stress response. Cell Death Differ. 28 (5), 1705–1719. 10.1038/s41418-020-00695-7 33288903 PMC8167164

[B91] Li X. Wang T. X. Huang X. Li Y. Sun T. Zang S. (2020). Targeting ferroptosis alleviates methionine-choline deficient (MCD)-diet induced NASH by suppressing liver lipotoxicity. Liver Int. 40 (6), 1378–1394. 10.1111/liv.14428 32145145

[B92] Li Y. L. Li X. Q. Wang Y. D. Shen C. Zhao C. Y. (2019). Metformin alleviates inflammatory response in non-alcoholic steatohepatitis by restraining signal transducer and activator of transcription 3-mediated autophagy inhibition *in vitro* and *in vivo* . Biochem. Biophys. Res. Commun. 513 (1), 64–72. 10.1016/j.bbrc.2019.03.077 30935688

[B93] Liang L. Ye S. Jiang R. Zhou X. Zhou J. Meng S. (2022). Liensinine alleviates high fat diet (HFD)-induced non-alcoholic fatty liver disease (NAFLD) through suppressing oxidative stress and inflammation via regulating TAK1/AMPK signaling. Int. Immunopharmacol. 104, 108306. 10.1016/j.intimp.2021.108306 34999396

[B94] Lim H. Lim Y. M. Kim K. H. Jeon Y. E. Park K. Kim J. (2018). A novel autophagy enhancer as a therapeutic agent against metabolic syndrome and diabetes. Nat. Commun. 9 (1), 1438. 10.1038/s41467-018-03939-w 29650965 PMC5897400

[B95] Lim J. S. Mietus-Snyder M. Valente A. Schwarz J. M. Lustig R. H. (2010). The role of fructose in the pathogenesis of NAFLD and the metabolic syndrome. Nat. Rev. Gastroenterol. Hepatol. 7 (5), 251–264. 10.1038/nrgastro.2010.41 20368739

[B96] Listenberger L. L. Han X. Lewis S. E. Cases S. Farese R. V. Jr. Ory D. S. (2003). Triglyceride accumulation protects against fatty acid-induced lipotoxicity. Proc. Natl. Acad. Sci. U. S. A. 100 (6), 3077–3082. 10.1073/pnas.0630588100 12629214 PMC152249

[B97] Liu J. Kuang F. Kroemer G. Klionsky D. J. Kang R. Tang D. (2020). Autophagy-dependent ferroptosis: machinery and regulation. Cell Chem. Biol. 27 (4), 420–435. 10.1016/j.chembiol.2020.02.005 32160513 PMC7166192

[B98] Liu J. Liu Y. Wang Y. Li C. Xie Y. Klionsky D. J. (2023). TMEM164 is a new determinant of autophagy-dependent ferroptosis. Autophagy 19 (3), 945–956. 10.1080/15548627.2022.2111635 35947500 PMC9980451

[B99] Liu Y. Song J. Yang J. Zheng J. Yang L. Gao J. (2021). Tumor necrosis factor α-induced protein 8-like 2 alleviates nonalcoholic fatty liver disease through suppressing transforming growth factor beta-activated kinase 1 activation. Hepatology 74 (3), 1300–1318. 10.1002/hep.31832 33768585

[B100] Loguercio C. De Girolamo V. de Sio I. Tuccillo C. Ascione A. Baldi F. (2001). Non-alcoholic fatty liver disease in an area of southern Italy: main clinical, histological, and pathophysiological aspects. J. Hepatol. 35 (5), 568–574. 10.1016/s0168-8278(01)00192-1 11690701

[B101] Madiraju A. K. Alves T. Zhao X. Cline G. W. Zhang D. Bhanot S. (2016). Argininosuccinate synthetase regulates hepatic AMPK linking protein catabolism and ureagenesis to hepatic lipid metabolism. Proc. Natl. Acad. Sci. U. S. A. 113 (24), E3423–E3430. 10.1073/pnas.1606022113 27247419 PMC4914193

[B102] Majdi A. Aoudjehane L. Ratziu V. Islam T. Afonso M. B. Conti F. (2020). Inhibition of receptor-interacting protein kinase 1 improves experimental non-alcoholic fatty liver disease. J. Hepatol. 72 (4), 627–635. 10.1016/j.jhep.2019.11.008 31760070

[B103] Malhi H. Kaufman R. J. (2011). Endoplasmic reticulum stress in liver disease. J. Hepatol. 54 (4), 795–809. 10.1016/j.jhep.2010.11.005 21145844 PMC3375108

[B104] Man S. M. Karki R. Kanneganti T. D. (2017). Molecular mechanisms and functions of pyroptosis, inflammatory caspases and inflammasomes in infectious diseases. Immunol. Rev. 277 (1), 61–75. 10.1111/imr.12534 28462526 PMC5416822

[B105] Marengo A. Jouness R. I. Bugianesi E. (2016). Progression and natural history of nonalcoholic fatty liver disease in adults. Clin. Liver Dis. 20 (2), 313–324. 10.1016/j.cld.2015.10.010 27063271

[B106] Marra F. Gastaldelli A. Svegliati Baroni G. Tell G. Tiribelli C. (2008). Molecular basis and mechanisms of progression of non-alcoholic steatohepatitis. Trends Mol. Med. 14 (2), 72–81. 10.1016/j.molmed.2007.12.003 18218340

[B107] Martinon F. Pétrilli V. Mayor A. Tardivel A. Tschopp J. (2006). Gout-associated uric acid crystals activate the NALP3 inflammasome. Nature 440 (7081), 237–241. 10.1038/nature04516 16407889

[B108] Mederacke I. Filliol A. Affo S. Nair A. Hernandez C. Sun Q. (2022). The purinergic P2Y14 receptor links hepatocyte death to hepatic stellate cell activation and fibrogenesis in the liver. Sci. Transl. Med. 14 (639), eabe5795. 10.1126/scitranslmed.abe5795 35385339 PMC9436006

[B109] Mejlvang J. Olsvik H. Svenning S. Bruun J. A. Abudu Y. P. Larsen K. B. (2018). Starvation induces rapid degradation of selective autophagy receptors by endosomal microautophagy. J. Cell Biol. 217 (10), 3640–3655. 10.1083/jcb.201711002 30018090 PMC6168274

[B110] Mirea A. M. Tack C. J. Chavakis T. Joosten L. A. B. Toonen E. J. M. (2018). IL-1 family cytokine pathways underlying NAFLD: towards new treatment strategies. Trends Mol. Med. 24 (5), 458–471. 10.1016/j.molmed.2018.03.005 29665983 PMC5939989

[B111] Morioka S. Broglie P. Omori E. Ikeda Y. Takaesu G. Matsumoto K. (2014). TAK1 kinase switches cell fate from apoptosis to necrosis following TNF stimulation. J. Cell Biol. 204 (4), 607–623. 10.1083/jcb.201305070 24535827 PMC3926964

[B112] Morioka S. Omori E. Kajino T. Kajino-Sakamoto R. Matsumoto K. Ninomiya-Tsuji J. (2009). TAK1 kinase determines TRAIL sensitivity by modulating reactive oxygen species and cIAP. Oncogene 28 (23), 2257–2265. 10.1038/onc.2009.110 19421137 PMC2796077

[B113] Murphy J. M. Czabotar P. E. Hildebrand J. M. Lucet I. S. Zhang J. G. Alvarez-Diaz S. (2013). The pseudokinase MLKL mediates necroptosis via a molecular switch mechanism. Immunity 39 (3), 443–453. 10.1016/j.immuni.2013.06.018 24012422

[B114] Nan Y. M. Wu W. J. Yao X. X. Wang L. (2007). The role of apoptosis and the related genes in non-alcoholic steatohepatitis. Zhonghua Gan Zang Bing Za Zhi 15 (1), 41–46. 10.3760/j.issn:1007-3418.2007.01.011 17244458

[B115] Nasiri-Ansari N. Nikolopoulou C. Papoutsi K. Kyrou I. Mantzoros C. S. Kyriakopoulos G. (2021). Empagliflozin attenuates non-alcoholic fatty liver disease (NAFLD) in high fat diet fed ApoE((-/-)) mice by activating autophagy and reducing ER stress and apoptosis. Int. J. Mol. Sci. 22 (2), 818. 10.3390/ijms22020818 33467546 PMC7829901

[B116] Oakes S. A. Papa F. R. (2015). The role of endoplasmic reticulum stress in human pathology. Annu. Rev. Pathol. 10, 173–194. 10.1146/annurev-pathol-012513-104649 25387057 PMC5568783

[B117] Oh C. J. Kim J. Y. Min A. K. Park K. G. Harris R. A. (2012). Sulforaphane attenuates hepatic fibrosis via NF-E2-related factor 2-mediated inhibition of transforming growth factor-β/Smad signaling. Free Radic. Biol. Med. 52 (3), 671–682. 10.1016/j.freeradbiomed.2011.11.012 22155056

[B118] Oku M. Sakai Y. (2018). Three distinct types of microautophagy based on membrane dynamics and molecular machineries. Bioessays 40 (6), e1800008. 10.1002/bies.201800008 29708272

[B119] Ouyang X. Cirillo P. Sautin Y. McCall S. Bruchette J. L. Diehl A. M. (2008). Fructose consumption as a risk factor for non-alcoholic fatty liver disease. J. Hepatol. 48 (6), 993–999. 10.1016/j.jhep.2008.02.011 18395287 PMC2423467

[B120] Pak E. Segal R. A. (2016). Hedgehog signal transduction: key players, oncogenic drivers, and cancer therapy. Dev. Cell 38 (4), 333–344. 10.1016/j.devcel.2016.07.026 27554855 PMC5017307

[B121] Pasparakis M. Vandenabeele P. (2015). Necroptosis and its role in inflammation. Nature 517 (7534), 311–320. 10.1038/nature14191 25592536

[B122] Pereira M. Chen T. D. Buang N. Olona A. Ko J. H. Prendecki M. (2019). Acute iron deprivation reprograms human macrophage metabolism and reduces inflammation *in vivo* . Cell Rep. 28 (2), 498–511. 10.1016/j.celrep.2019.06.039 31291584 PMC6635384

[B123] Preston S. P. Stutz M. D. Allison C. C. Nachbur U. Gouil Q. Tran B. M. (2022). Epigenetic silencing of RIPK3 in hepatocytes prevents MLKL-mediated necroptosis from contributing to liver pathologies. Gastroenterology 163 (6), 1643–1657.e14. 10.1053/j.gastro.2022.08.040 36037995

[B124] Qi J. Kim J. W. Zhou Z. Lim C. W. Kim B. (2020). Ferroptosis affects the progression of nonalcoholic steatohepatitis via the modulation of lipid peroxidation-mediated cell death in mice. Am. J. Pathol. 190 (1), 68–81. 10.1016/j.ajpath.2019.09.011 31610178

[B125] Rinella M. E. Sookoian S. (2023). From NAFLD to MASLD: updated naming and diagnosis criteria for fatty liver disease. J. Lipid Res. 65 (1), 100485. 10.1016/j.jlr.2023.100485 38103785 PMC10824973

[B126] Rogers C. Fernandes-Alnemri T. Mayes L. Alnemri D. Cingolani G. (2017). Cleavage of DFNA5 by caspase-3 during apoptosis mediates progression to secondary necrotic/pyroptotic cell death. Nat. Commun. 8, 14128. 10.1038/ncomms14128 28045099 PMC5216131

[B127] Safari Z. Gérard P. (2019). The links between the gut microbiome and non-alcoholic fatty liver disease (NAFLD). Cell Mol. Life Sci. 76 (8), 1541–1558. 10.1007/s00018-019-03011-w 30683985 PMC11105223

[B128] Schock S. N. Chandra N. V. Sun Y. Irie T. Kitagawa Y. Gotoh B. (2017). Induction of necroptotic cell death by viral activation of the RIG-I or STING pathway. Cell Death Differ. 24 (4), 615–625. 10.1038/cdd.2016.153 28060376 PMC5384020

[B129] Sharma B. R. Kanneganti T. D. (2021). NLRP3 inflammasome in cancer and metabolic diseases. Nat. Immunol. 22 (5), 550–559. 10.1038/s41590-021-00886-5 33707781 PMC8132572

[B130] Shen T. Lei T. Chen L. Zhu B. B. Xu B. L. Zhang C. P. (2021). Gardenoside hinders caspase-1-mediated hepatocyte pyroptosis through the CTCF/DPP4 signaling pathway. Front. Physiol. 12, 669202. 10.3389/fphys.2021.669202 34566670 PMC8455910

[B131] Shimizu S. Kanaseki T. Mizushima N. Mizuta T. Arakawa-Kobayashi S. Thompson C. B. (2004). Role of Bcl-2 family proteins in a non-apoptotic programmed cell death dependent on autophagy genes. Nat. Cell Biol. 6 (12), 1221–1228. 10.1038/ncb1192 15558033

[B132] Shimizu S. Yoshida T. Tsujioka M. Arakawa S. (2014). Autophagic cell death and cancer. Int. J. Mol. Sci. 15 (2), 3145–3153. 10.3390/ijms15023145 24566140 PMC3958902

[B133] Singh R. Kaushik S. Wang Y. Xiang Y. Novak I. Komatsu M. (2009). Autophagy regulates lipid metabolism. Nature 458 (7242), 1131–1135. 10.1038/nature07976 19339967 PMC2676208

[B134] Smith B. K. Marcinko K. Desjardins E. M. Lally J. S. Ford R. J. Steinberg G. R. (2016). Treatment of nonalcoholic fatty liver disease: role of AMPK. Am. J. Physiol. Endocrinol. Metab. 311 (4), E730–e740. 10.1152/ajpendo.00225.2016 27577854

[B135] Softic S. Cohen D. E. Kahn C. R. (2016). Role of dietary fructose and hepatic *de novo* lipogenesis in fatty liver disease. Dig. Dis. Sci. 61 (5), 1282–1293. 10.1007/s10620-016-4054-0 26856717 PMC4838515

[B136] Song X. Zhu S. Chen P. Hou W. Wen Q. Liu J. (2018). AMPK-mediated BECN1 phosphorylation promotes ferroptosis by directly blocking system X(c)(-) activity. Curr. Biol. 28 (15), 2388–2399. 10.1016/j.cub.2018.05.094 30057310 PMC6081251

[B137] Sun L. Wang H. Wang Z. He S. Chen S. Liao D. (2012). Mixed lineage kinase domain-like protein mediates necrosis signaling downstream of RIP3 kinase. Cell 148 (1-2), 213–227. 10.1016/j.cell.2011.11.031 22265413

[B138] Syn W. K. Choi S. S. Liaskou E. Karaca G. F. Agboola K. M. Oo Y. H. (2011). Osteopontin is induced by hedgehog pathway activation and promotes fibrosis progression in nonalcoholic steatohepatitis. Hepatology 53 (1), 106–115. 10.1002/hep.23998 20967826 PMC3025083

[B139] Tekirdag K. Cuervo A. M. (2018). Chaperone-mediated autophagy and endosomal microautophagy: joint by a chaperone. J. Biol. Chem. 293 (15), 5414–5424. 10.1074/jbc.R117.818237 29247007 PMC5900761

[B140] Tilg H. Moschen A. R. (2010). Evolution of inflammation in nonalcoholic fatty liver disease: the multiple parallel hits hypothesis. Hepatology 52 (5), 1836–1846. 10.1002/hep.24001 21038418

[B141] Tricarico P. M. Marcuzzi A. Piscianz E. Monasta L. Crovella S. Kleiner G. (2013). Mevalonate kinase deficiency and neuroinflammation: balance between apoptosis and pyroptosis. Int. J. Mol. Sci. 14 (12), 23274–23288. 10.3390/ijms141223274 24287904 PMC3876043

[B142] Tsvetkov P. Coy S. Petrova B. Dreishpoon M. Verma A. Abdusamad M. (2022). Copper induces cell death by targeting lipoylated TCA cycle proteins. Science 375 (6586), 1254–1261. 10.1126/science.abf0529 35298263 PMC9273333

[B143] Upton J. W. Kaiser W. J. Mocarski E. S. (2019). DAI/ZBP1/DLM-1 complexes with RIP3 to mediate virus-induced programmed necrosis that is targeted by murine cytomegalovirus vIRA. Cell Host Microbe 26 (4), 564. 10.1016/j.chom.2019.09.004 31600504

[B144] Van Cruchten S. Van Den Broeck W. (2002). Morphological and biochemical aspects of apoptosis, oncosis and necrosis. Anat. Histol. Embryol. 31 (4), 214–223. 10.1046/j.1439-0264.2002.00398.x 12196263

[B145] Vivoli E. Cappon A. Milani S. Piombanti B. Provenzano A. Novo E. (2016). NLRP3 inflammasome as a target of berberine in experimental murine liver injury: interference with P2X7 signalling. Clin. Sci. (Lond) 130 (20), 1793–1806. 10.1042/CS20160400 27439970

[B146] Volkmann X. Fischer U. Bahr M. J. Ott M. Lehner F. Macfarlane M. (2007). Increased hepatotoxicity of tumor necrosis factor-related apoptosis-inducing ligand in diseased human liver. Hepatology 46 (5), 1498–1508. 10.1002/hep.21846 17705261

[B147] Vucur M. Ghallab A. Schneider A. T. Adili A. Cheng M. Castoldi M. (2023). Sublethal necroptosis signaling promotes inflammation and liver cancer. Immunity 56, 1578–1595.e8. 10.1016/j.immuni.2023.05.017 37329888

[B148] Wali J. A. Rondas D. McKenzie M. D. Zhao Y. Elkerbout L. Fynch S. (2014). The proapoptotic BH3-only proteins Bim and Puma are downstream of endoplasmic reticulum and mitochondrial oxidative stress in pancreatic islets in response to glucotoxicity. Cell Death Dis. 5 (3), e1124. 10.1038/cddis.2014.88 24625983 PMC3973197

[B149] Wang C. Y. Mayo M. W. Korneluk R. G. Goeddel D. V. Baldwin A. S. Jr. (1998). NF-kappaB antiapoptosis: induction of TRAF1 and TRAF2 and c-IAP1 and c-IAP2 to suppress caspase-8 activation. Science 281 (5383), 1680–1683. 10.1126/science.281.5383.1680 9733516

[B150] Wang K. Sun Q. Zhong X. Zeng M. Zeng H. Shi X. (2020). Structural mechanism for GSDMD targeting by autoprocessed caspases in pyroptosis. Cell 180 (5), 941–955. 10.1016/j.cell.2020.02.002 32109412

[B151] Wang P. X. Zhang X. J. Luo P. Jiang X. Zhang P. Guo J. (2016b). Hepatocyte TRAF3 promotes liver steatosis and systemic insulin resistance through targeting TAK1-dependent signalling. Nat. Commun. 7, 10592. 10.1038/ncomms10592 26882989 PMC4757796

[B152] Wang S. Luo J. Zhang Z. Dong D. Shen Y. Fang Y. (2018a). Iron and magnetic: new research direction of the ferroptosis-based cancer therapy. Am. J. Cancer Res. 8 (10), 1933–1946. 30416846 PMC6220147

[B153] Wang W. Gao W. Zhu Q. Alasbahi A. Seki E. Yang L. (2021). TAK1: a molecular link between liver inflammation, fibrosis, steatosis, and carcinogenesis. Front. Cell Dev. Biol. 9, 734749. 10.3389/fcell.2021.734749 34722513 PMC8551703

[B154] Wang X. Zheng Z. Caviglia J. M. Corey K. E. Herfel T. M. Cai B. (2016a). Hepatocyte TAZ/WWTR1 promotes inflammation and fibrosis in nonalcoholic steatohepatitis. Cell Metab. 24 (6), 848–862. 10.1016/j.cmet.2016.09.016 28068223 PMC5226184

[B155] Wang Z. Yin W. Zhu L. Li J. Yao Y. Chen F. (2018b). Iron drives T helper cell pathogenicity by promoting RNA-binding protein PCBP1-mediated proinflammatory cytokine production. Immunity 49 (1), 80–92. 10.1016/j.immuni.2018.05.008 29958803

[B156] Witek R. P. Stone W. C. Karaca F. G. Syn W. K. Pereira T. A. Agboola K. M. (2009). Pan-caspase inhibitor VX-166 reduces fibrosis in an animal model of nonalcoholic steatohepatitis. Hepatology 50 (5), 1421–1430. 10.1002/hep.23167 19676126

[B157] Wu J. Sun J. Meng X. (2021a). Pyroptosis by caspase-11 inflammasome-Gasdermin D pathway in autoimmune diseases. Pharmacol. Res. 165, 105408. 10.1016/j.phrs.2020.105408 33412278

[B158] Wu S. Yang J. Sun G. Hu J. Zhang Q. Cai J. (2021b). Macrophage extracellular traps aggravate iron overload-related liver ischaemia/reperfusion injury. Br. J. Pharmacol. 178 (18), 3783–3796. 10.1111/bph.15518 33959955

[B159] Wu X. Poulsen K. L. Sanz-Garcia C. Huang E. McMullen M. R. Roychowdhury S. (2020). MLKL-dependent signaling regulates autophagic flux in a murine model of non-alcohol-associated fatty liver and steatohepatitis. J. Hepatol. 73 (3), 616–627. 10.1016/j.jhep.2020.03.023 32220583 PMC7438259

[B160] Xia X. Wang X. Zheng Y. Jiang J. Hu J. (2019). What role does pyroptosis play in microbial infection? J. Cell Physiol. 234 (6), 7885–7892. 10.1002/jcp.27909 30537070

[B161] Xu B. Jiang M. Chu Y. Wang W. Chen D. Li X. (2018). Gasdermin D plays a key role as a pyroptosis executor of non-alcoholic steatohepatitis in humans and mice. J. Hepatol. 68 (4), 773–782. 10.1016/j.jhep.2017.11.040 29273476

[B162] Yamada K. Mizukoshi E. Sunagozaka H. Arai K. Yamashita T. Takeshita Y. (2015). Characteristics of hepatic fatty acid compositions in patients with nonalcoholic steatohepatitis. Liver Int. 35 (2), 582–590. 10.1111/liv.12685 25219574

[B163] Yang D. He Y. Muñoz-Planillo R. Liu Q. Núñez G. (2015). Caspase-11 requires the pannexin-1 channel and the purinergic P2X7 pore to mediate pyroptosis and endotoxic shock. Immunity 43 (5), 923–932. 10.1016/j.immuni.2015.10.009 26572062 PMC4795157

[B164] Yang L. Roh Y. S. Song J. Zhang B. Liu C. Loomba R. (2014). Transforming growth factor beta signaling in hepatocytes participates in steatohepatitis through regulation of cell death and lipid metabolism in mice. Hepatology 59 (2), 483–495. 10.1002/hep.26698 23996730 PMC3946696

[B165] Yang Y. Chen J. Gao Q. Shan X. Wang J. Lv Z. (2020). Study on the attenuated effect of Ginkgolide B on ferroptosis in high fat diet induced nonalcoholic fatty liver disease. Toxicology 445, 152599. 10.1016/j.tox.2020.152599 32976958

[B166] Yang Z. Wu J. Li X. Xie D. Wang Y. Yang T. (2019). Association between dietary iron intake and the prevalence of nonalcoholic fatty liver disease: a cross-sectional study. Med. Baltim. 98 (43), e17613. 10.1097/MD.0000000000017613 PMC6824640 31651873

[B167] Younossi Z. Anstee Q. M. Marietti M. Hardy T. Henry L. Eslam M. (2018). Global burden of NAFLD and NASH: trends, predictions, risk factors and prevention. Nat. Rev. Gastroenterol. Hepatol. 15 (1), 11–20. 10.1038/nrgastro.2017.109 28930295

[B168] Yu X. Hao M. Liu Y. Ma X. Lin W. Xu Q. (2019). Liraglutide ameliorates non-alcoholic steatohepatitis by inhibiting NLRP3 inflammasome and pyroptosis activation via mitophagy. Eur. J. Pharmacol. 864, 172715. 10.1016/j.ejphar.2019.172715 31593687

[B169] Zhang H. Zhang E. Hu H. (2021a). Role of ferroptosis in non-alcoholic fatty liver disease and its implications for therapeutic strategies. Biomedicines 9 (11), 1660. 10.3390/biomedicines9111660 34829889 PMC8615581

[B170] Zhang M. Wu P. Li M. Guo Y. Tian T. Liao X. (2021b). Inhibition of Notch1 signaling reduces hepatocyte injury in nonalcoholic fatty liver disease via autophagy. Biochem. Biophys. Res. Commun. 547, 131–138. 10.1016/j.bbrc.2021.02.039 33610041

[B171] Zhao H. Liu H. Yang Y. Wang H. (2022). The role of autophagy and pyroptosis in liver disorders. Int. J. Mol. Sci. 23 (11), 6208. 10.3390/ijms23116208 35682887 PMC9181643

[B172] Zhao P. Sun X. Chaggan C. Liao Z. He F. Wong K. (2020). An AMPK-caspase-6 axis controls liver damage in nonalcoholic steatohepatitis. Science 367 (6478), 652–660. 10.1126/science.aay0542 32029622 PMC8012106

[B173] Zhong Y. Wang J. J. Zhang S. X. (2012). Intermittent but not constant high glucose induces ER stress and inflammation in human retinal pericytes. Adv. Exp. Med. Biol. 723, 285–292. 10.1007/978-1-4614-0631-0_37 22183344 PMC3243941

[B174] Zhou B. Zhang J. Y. Liu X. S. Chen H. Z. Ai Y. L. Cheng K. (2018). Tom20 senses iron-activated ROS signaling to promote melanoma cell pyroptosis. Cell Res. 28 (12), 1171–1185. 10.1038/s41422-018-0090-y 30287942 PMC6274649

[B175] Zhu C. Kim K. Wang X. Bartolome A. Salomao M. Dongiovanni P. (2018). Hepatocyte Notch activation induces liver fibrosis in nonalcoholic steatohepatitis. Sci. Transl. Med. 10 (468), eaat0344. 10.1126/scitranslmed.aat0344 30463916 PMC6822168

[B176] Zhu J. Wen Y. Zhang Q. Nie F. Cheng M. Zhao X. (2022). The monomer TEC of blueberry improves NASH by augmenting tRF-47-mediated autophagy/pyroptosis signaling pathway. J. Transl. Med. 20 (1), 128. 10.1186/s12967-022-03343-5 35287671 PMC8919551

[B177] Zhu Z. Zhang Y. Huang X. Can L. Zhao X. Wang Y. (2021). Thymosin beta 4 alleviates non-alcoholic fatty liver by inhibiting ferroptosis via up-regulation of GPX4. Eur. J. Pharmacol. 908, 174351. 10.1016/j.ejphar.2021.174351 34280397

